# Central Role of the Holliday Junction Helicase RuvAB in *vlsE* Recombination and Infectivity of *Borrelia burgdorferi*

**DOI:** 10.1371/journal.ppat.1000679

**Published:** 2009-12-04

**Authors:** Tao Lin, Lihui Gao, Diane G. Edmondson, Mary B. Jacobs, Mario T. Philipp, Steven J. Norris

**Affiliations:** 1 Department of Pathology and Laboratory Medicine, Medical School, University of Texas Health Science Center at Houston, Houston, Texas, United States of America; 2 Division of Bacteriology and Parasitology, Tulane National Primate Research Center, Tulane University Health Sciences Center, Covington, Louisiana, United States of America; 3 Department of Microbiology and Molecular Genetics, Medical School, University of Texas Health Science Center at Houston, Houston, Texas, United States of America; Medical College of Wisconsin, United States of America

## Abstract

Antigenic variation plays a vital role in the pathogenesis of many infectious bacteria and protozoa including *Borrelia burgdorferi*, the causative agent of Lyme disease. VlsE, a 35 kDa surface-exposed lipoprotein, undergoes antigenic variation during *B. burgdorferi* infection of mammalian hosts, and is believed to be a critical mechanism by which the spirochetes evade immune clearance. Random, segmental recombination between the expressed *vlsE* gene and adjacent *vls* silent cassettes generates a large number of different VlsE variants within the infected host. Although the occurrence and importance of *vlsE* sequence variation is well established, little is known about the biological mechanism of *vlsE* recombination. To identify factors important in antigenic variation and *vlsE* recombination, we screened transposon mutants of genes known to be involved in DNA recombination and repair for their effects on infectivity and *vlsE* recombination. Several mutants, including those in BB0023 (*ruvA*), BB0022 (*ruvB*), BB0797 (*mutS*), and BB0098 (*mutS-II*), showed reduced infectivity in immunocompetent C3H/HeN mice. Mutants in *ruvA* and *ruvB* exhibited greatly reduced rates of *vlsE* recombination in C3H/HeN mice, as determined by restriction fragment polymorphism (RFLP) screening and DNA sequence analysis. In severe combined immunodeficiency (C3H/*scid*) mice, the *ruvA* mutant retained full infectivity; however, all recovered clones retained the ‘parental’ *vlsE* sequence, consistent with low rates of *vlsE* recombination. These results suggest that the reduced infectivity of *ruvA* and *ruvB* mutants is the result of ineffective *vlsE* recombination and underscores the important role that *vlsE* recombination plays in immune evasion. Based on functional studies in other organisms, the RuvAB complex of *B. burgdorferi* may promote branch migration of Holliday junctions during *vlsE* recombination. Our findings are consistent with those in the accompanying article by Dresser et al., and together these studies provide the first examples of trans-acting factors involved in *vlsE* recombination.

## Author Summary

Lyme disease is the most prevalent tick-borne infection in North America and Eurasia. It is caused by the bacterium *Borrelia burgdorferi* and is transmitted to humans via the bite of infected ticks. These spirochetes can cause both acute and chronic infection and inflammation of the skin, joints, heart, and central nervous system. The persistence of infection despite the presence of an active immune response is dependent upon antigenic variation of VlsE, a 35 kDa surface-exposed lipoprotein. A large number of different VlsE variants are present in the host simultaneously and are generated by recombination of the *vlsE* gene with adjacent *vls* silent cassettes. To try to identify factors important in *vlsE* recombination and immune evasion, we selected mutants in genes involved in DNA recombination and repair and screened them for infectivity and *vlsE* recombination. Mutants in genes encoding RuvA and RuvB (which act together to promote the exchange of strands between two different DNA molecules) had reduced infectivity and greatly diminished *vlsE* recombination. In immunodeficient mice, *ruvA* mutants retained full infectivity, and no *vlsE* recombination was detected. Our findings reinforce the importance of *vlsE* variation in immune evasion and persistent infection.

## Introduction

Lyme borreliosis is a multi-stage, systemic disease caused by members of the spirochete genus *Borrelia*, including *Borrelia burgdorferi* in North America and *Borrelia afzelii*, *Borrelia garinii*, and *B. burgdorferi* in Eurasia [1]. Spirochetes are transmitted to mammalian and avian hosts via the bite of hard-bodied ticks of *Ixodes* genus [2] and disseminate widely throughout the body in the first weeks of infection. If untreated in early stages, chronic and debilitating disease can develop in the skin, joints, heart, and central nervous system [1]. Infected individuals develop an active immune response to the pathogen yet are unable to clear the infection.

A common mechanism of immune evasion is antigenic variation, a process by which pathogens alter surface exposed antigenic proteins [3],[4]. The resulting variant organisms are immunologically distinct from parental strains and thereby gain a selective advantage over individuals that retain parental antigenic determinants. Bacteria that undergo antigenic variation often cause long term or repeated infections. Examples of such bacteria include *Neisseria gonorrhoeae*, *Neisseria meningitidis*, *Borrelia hermsii*, *Treponema pallidum*, *Campylobacter jejuni*, *Mycoplasma synoviae*, *Mycoplasma pulmonis*, and *Anaplasma marginale*.

Lyme disease *Borrelia* possess the *vls* (**V**ariable Major Protein (VMP)-**l**ike **s**equence) system, a robust antigenic variation mechanism involving DNA recombination at the locus that expresses the surface exposed lipoprotein VlsE [5],[6],[7],[8],[9]. The *vls* locus consists of the *vlsE* expression site and a contiguous array of 15 *vls* silent cassettes, which have homology to the central region of the expression site. Gene conversion events involving replacement of regions of the *vlsE* expression site cassette with segments of the silent cassettes occur continuously during mouse infection, resulting in a myriad of *vlsE* sequence variants in each infected animal [10],[11]. Within each cassette, there are six variable regions (VRs) that display considerable sequence diversity, interspersed with six relatively invariant regions (IRs). The structure of the VlsE polypeptide is predominated by alpha helices that are believed to be important in maintaining protein structure [12]. The variable regions form random coils on the membrane distal surface of the protein, the region most likely to be exposed to the host immune system. VlsE variants have different epitopes when compared with parental VlsE1 polypeptide [10],[13], indicating that sequence changes in *vlsE* result in true antigenic variation.

In the *B. burgdorferi* strain B31, the *vls* locus is located near the telomere of the linear plasmid lp28-1 [10]. Loss of lp28-1 in *B. burgdorferi* B31 is associated with an intermediate infectivity phenotype in immunocompetent mice, in which infection lasts for less than 3 weeks and is largely restricted to joint tissue [14],[15],[16],[17]. Spirochetes lacking lp28-1 are able to survive and cause disease at all tissue sites in severe combined immunodeficiency (SCID) mice [17],[18], indicating that one or more gene products encoded on lp28-1 play an important protective role against adaptive immunity. Bankhead and Chaconas [19] recently demonstrated that the *vls* locus is the important mediator of infectivity in lp28-1. Deletion of the locus by telomere-mediated truncation resulted in an intermediate infectivity phenotype similar to that displayed by *B. burgdorferi* lacking lp28-1, while deletion of the other end of the plasmid had no detectable effect on infectivity.

The mechanisms that regulate *vlsE* recombination and expression are not well understood. *vlsE* recombination events have not been detected in ticks, during in vitro culture, or in dialysis membrane chambers embedded in rats, suggesting that non-dialyzable host components play a role in initiation of recombination [20],[21],[22],[23]. In contrast, *vlsE* recombination is detectable within 4 days of mouse inoculation, and appears to occur throughout the course of mammalian infection [22],[24]. Approximately 50% of recovered spirochetes have recombined *vlsE* sequences during the first 7 days of mouse infection; by four weeks, no organisms with parental *vlsE* sequence can be recovered [11],[22]. In immunodeficient SCID mice, spirochetes with the parental *vlsE* disappear more gradually, indicating that the adaptive immune response is required for the rapid elimination of organisms expressing the parental VlsE [11]. Although analyses of *vlsE* transcription levels during mammalian infection have yielded variable results, immunoblot analysis indicates that VlsE protein expression is dramatically upregulated during mouse or rabbit infection [25],[26].

Genes involved in DNA recombination and repair have been found to play a role in antigenic variation in other bacteria. For example, in *Neisseria gonorrhoeae*, pilin antigenic variation requires several trans-acting factors in the RecF-like recombination pathway, including RecA, RecX, RecJ, RecO, RecQ, and RecR [27],[28],[29] as well as RecG and a functional RuvABC complex [30]. However, in *B. burgdorferi* no genes have been yet identified that play a role in recombination within *vlsE*, although *recA* has been shown to be dispensable [31].

To identify proteins that play a role in *vlsE* recombination, we screened transposon mutants of DNA recombination and repair genes for infectivity and *vlsE* recombination. We found that mutants in the genes encoding the Holliday junction helicase polypeptides RuvA and RuvB were compromised in their ability to undergo *vlsE* recombination and displayed a reduced infectivity phenotype. In SCID mice, *ruvA* mutants retained full infectivity. *vlsE* sequences recovered from SCID mice were identical to parental *vlsE* sequence, suggesting that the failure to undergo *vlsE* recombination was a critical factor in the reduced infectivity phenotype. Furthermore, *ruvA* mutants had no increase in sensitivity to DNA damage. Nearly identical results were obtained in the accompanying article by Dresser et al. [32].

## Results

### Screening of putative DNA recombination and repair mutants for infectivity and VlsE variation

For this study, 9 transposon insertion mutants representing 7 different genes whose homologues are known to be involved in DNA recombination and repair in other bacteria were selected from a large Signature Tagged Mutagenesis (STM) panel to screen for their effect on infectivity and *vlsE* recombination (Table 1). Independent mutants from two of the genes were included to test the possible role of different insertion sites on infectivity and *vlsE* recombination. T05P01C02 and T10P01D06 are independent mutants of BB0797 with insertion ratios of 0.73 and 0.97 respectively. (The insertion ratio represents the relative position of the transposon insertion in a coding sequence, with the entire coding sequence having a value of 1.0). Likewise, T08P01E02 and T11P01F09 are independent mutants from gene BBE29 with insertion ratios of 0.33 and 0.30. Each mutant was injected in 5 mice and skin biopsies were taken at days 7 and 14 after inoculation and cultured for *B. burgdorferi*. Mice were sacrificed on day 28 of infection, and samples taken from skin, tibiotarsal joint, heart, and bladder were cultured to recover infectious organisms. *B. burgdorferi* maintained in in vitro culture sometimes lose plasmids that are not required for in vitro growth but may be required for infectivity. Therefore, the plasmid content of each mutant was determined and compared with that of the parental clone 5A18NP1. Circular plasmid cp9 was found to be missing in two mutants (T11P01A01 and T11P01F09) and lp21 is lacking in T10P01D09. However, previous studies have shown that neither cp9 nor lp21 are essential for infection of mice [14],[15].

**Table 1:** Transposon mutants utilized in this study.

Mutant	Replicon	Insertion Site^a^	Insertion Ratio^b^	Gene	Predicted Product	Infectivity	Results, RFLP Analysis of *vlsE* recombination
T11P01A01	Chrm	22339	0.67	BB0023	junction DNA helicase, subunit A (RuvA)	Reduced	Reduced
T03TC051	Chrm	21916	0.18	BB0022	junction DNA helicase, subunit B (RuvB)	Reduced	Reduced
T05P01C02	Chrm	841133	0.73	BB0797	DNA mismatch repair protein (MutS)	Reduced	Reduced
T10P01D06	Chrm	841754	0.97	BB0797	DNA mismatch repair protein (MutS)	Normal	Normal
T10P01G11	Chrm	632960	0.24	BB0607	rep helicase, single-stranded DNA-dependent ATPase (Rep)	Normal	Normal
MG065	Chrm	96742	0.09	BB0098	recombination and DNA strand exchange inhibitor protein (MutS-II)	Noninfectious	ND^c^
T08P01E02	lp25	20092	0.33	BBE29	adenine specific DNA methyltransferase, authentic frameshift	Normal	Normal
T11P01F09	lp25	20047	0.30	BBE29	adenine specific DNA methyltransferase, authentic frameshift	Normal	ND
T09P01G01	lp17	12892	0.88	BBD20	transposase-like protein, authentic frameshift	Normal	Normal

The plasmid content of the mutants was the same as that of the parent strain 5A18NP1 (lp28-4^−^, lp56^−^) for all of the clones except for T11P01A01 and T11P01F09, which were also cp9^−^.

a Coordinates of transposon insertion in the indicated replicon. Chrm = chromosome.

b Number of nucleotides(beginning of the open reading frame to the transposon insertion site)/Number of nucleotides(open reading frame).

c Not determined.

As in previous studies, the parental strain 5A18NP1 was recovered from 29 of 30 tissue samples on days 7, 14, and 28 post inoculation [33]. Three of the nine DNA repair mutants tested (T11P01A01, T03TC051, and T05P01C02) exhibited reduced infectivity and one (MG065) lost infectivity in immunocompetent mice (Table 2). One of the *mutS* mutants (T05P01C02) displayed a reduced infectivity phenotype, whereas the other mutant (T10P01D06) had normal infectivity. The insertion site for the highly infectious mutant was very close to the end of the coding sequence (0.97), so this mutant may express a functional product. *ruvA* (T11P01A01) and *ruvB* (T03TC051) mutants both exhibited intermediate infectivity patterns. While 5/5 skin cultures were positive for both mutants at day 7 post inoculation, by 2 weeks no skin cultures were spirochete positive, and at 4 weeks only 6 of 20 and 10 of 16 tissue sites were culture positive for the *ruvA* and *ruvB* mutants, respectively. We also examined the growth rates of the *ruvA* and *ruvB* mutants in vitro. Both mutants had growth kinetics very similar to that of the parental strain 5A18NP1 (data not shown), indicating that the reduced infectivity phenotype observed for the mutants was not due to a growth defect.

**Table 2:** Infectivity of *B. burgdorferi* DNA repair and recombination mutants in CH3/HeN mice at days 7, 14 and 28 post inoculation.

				Day 7	Day 14	Day 28					
				No. of cultures positive/total							No. of mice positive/total
Strain	Gene disrupted	Gene Name	Insertion Ratio	Skin	Skin	Skin	Joint	Heart	Bladder	All sites	
5A18NP1 (control)	–	–	–	5/5	5/5	4/5	5/5	5/5	5/5	19/20	5/5
T11P01A01	BB0023	*ruvA*	0.67	5/5	0/5	2/5	2/5	1/5	1/5	6/20	3/5
T03TC051	BB0022	*ruvB*	0.18	4/4	0/4	3/4	3/4	2/4	2/4	10/16	3/4
T05P01C02	BB0797	*mutS*	0.73	1/5	1/5	1/5	1/5	1/5	1/5	4/20	1/5
T10P01D06	BB0797	*mutS*	0.97	5/5	5/5	4/5	5/5	5/5	5/5	19/20	5/5
T10P01G11	BB0607	*rep*	0.24	5/5	5/5	5/5	5/5	5/5	5/5	20/20	5/5
MG065	BB0098	*mutS-II*	0.09	0/5	0/5	0/5	0/5	0/5	0/5	0/20	0/5
T08P01E02	BBE29	--a	0.33	5/5	5/5	5/5	5/5	5/5	5/5	20/20	5/5
T11P01F09	BBE29	--a	0.30	5/5	5/5	5/5	5/5	5/5	5/5	20/20	5/5
T09P01G01	BBD20	--b	0.88	5/5	5/5	3/5	5/5	5/5	5/5	18/20	5/5

Each of 4 or 5 mice per group were inoculated intradermally at the base of the tail with 10^5^ of the indicated *B. burgdorferi* strain.

a adenine specific DNA methyltransferase, authentic frameshift.

b transposase-like protein, authentic frameshift.

**Figure 1:**
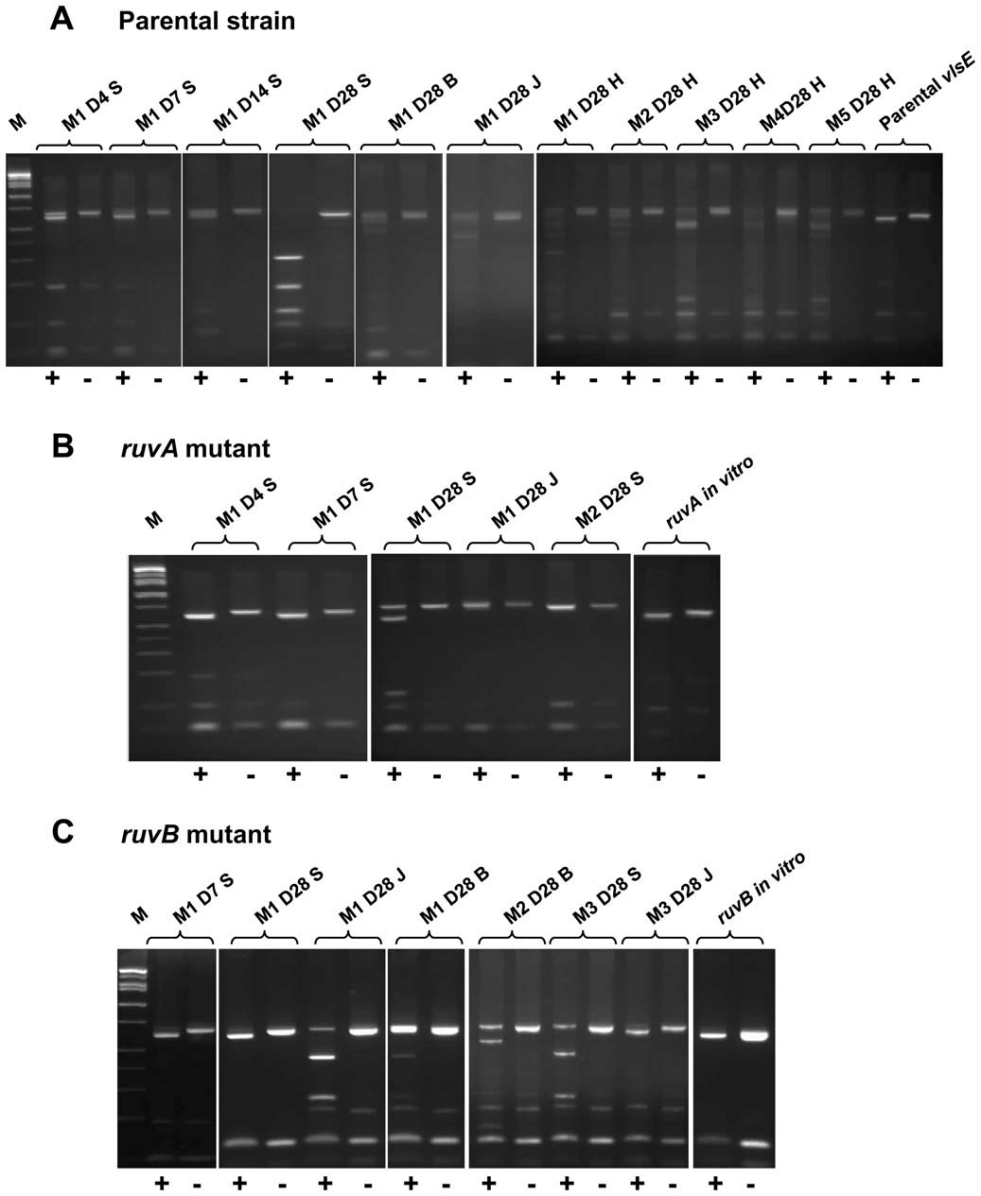
RFLP analysis of *vlsE* cassette region PCR products indicates reduced *vlsE* variation in *ruvA* and *ruvB* mutants of *B. burgdorferi*. Representative results obtained following inoculation of C3H/HeN mice with (A) the parental clone 5A18NP1, (B) the transposon mutant T11P01A01 (*ruvA::himar1*) and (C) T03TC051 (*ruvB::himar1*) are shown. Cultures from the indicated time points and tissues were used as the source of template DNA for amplification of the *vlsE* cassette region. The resulting PCR products were either treated (+) or not treated (-) with the restriction enzyme *Hph*I. Presence of multiple bands or a smear in the *Hph*I-treated sample is indicative of the presence of a high number of *vlsE* variants. The day post inoculation is indicated by D4 through D28. Tissues examined include skin (S), bladder (B), heart (H) and tibiotarsal joint (J).

Restriction fragment length polymorphism (RFLP) was used to estimate the extent of *vlsE* variation in recovered organisms. Amplicons corresponding to the *vlsE* cassette region were generated by PCR from uncloned cultures and digested with *Hph*I. This restriction enzyme recognizes a 4-bp sequence that occurs with varying frequency as a result of *vlsE* recombination. The *vlsE* cassette region of 5A18NP1, the parental strain used in this study, has a single *Hph*I site near one end (Figure 1A). As expected, cultures from mice inoculated with 5A18NP1 displayed significant RFLP variability that increased over the course of infection, as indicated by multiple bands or the presence of a smear in the *Hph*I-digested samples (Figure 1A). In contrast, the *ruvA* and *ruvB* mutant cultures (Figure 1B and 1C) had *vlsE* RFLP patterns that were either unchanged or had relatively few bands without smearing, indicating that few *vlsE* variants had been generated during the course of infection. The other putative DNA recombination and repair mutants had complex *vlsE* RFLP patterns, indicating that a variety of *vlsE* variants had been generated during infection and that *vlsE* recombination likely had occurred normally (data not shown).

### Characterization of the *ruvA* mutant

*ruvA* and *ruvB* have been reported to be part of a larger operon containing two additional genes (Figure S1A), *queA* (BB0021, S-adenosylmethionine:tRNA ribosyltransferase-isomerase) and *pfpB* (BB0020, diphosphate-fructose-6-phosphate 1-phosphotransferase) [34]. To determine whether expression of other genes in the operon might be disturbed by the transposon insertion, we performed RT-PCR on RNA samples from the parental strain 5A18NP1 and the *ruvA* mutant T11P01A01. Primer pair 1 generates a PCR fragment from within the *ruvA* coding region that crosses the transposon insertion site. As expected, this fragment can be generated using RNA from the parental strain, but not using RNA from the *ruvA* mutant, or RNA untreated with reverse transcriptase (Figure S1B). However, RT-PCR products spanning regions of both *ruvB* and *queA* were generated from both strains using primer pair 3 and 4 (Figure S1B). In the *ruvA* mutant T11P01A01, the transposon is inserted such that a ‘read-through’ transcript could possibly be generated from the *flgB_P_::aacC1* cassette within the transposable element. To test that possibility we used primer pair 2, which generates a PCR fragment corresponding to the *ruvA* coding sequence downstream of the transposon insertion site. A product could be generated from both strains (Figure S1B), suggesting that ‘read-through’ may be occurring from either the transposon or the native promoter. Thus, we conclude that T11P01A01 is defective in expression of *ruvA*, but that transcription of *ruvB*, *queA* and *pfpB* is occurring. However, we cannot rule out the possibility of polar effects resulting from changes in the levels of expression of *ruvB*, *queA* and *pfpB*.

### Inactivation of *ruvA* results in reduced infectivity in immunocompetent (C3H/HeN) mice but full infectivity in immunodeficient (SCID) mice

To further examine the effects of *ruvA* disruption on infectivity and *vlsE* recombination, a more detailed analysis of infectivity was performed (Table 3). Groups of ten immunocompetent C3H/HeN mice and ten C3H/*scid* mice were each inoculated with 10^5^ organisms of either the T11P01A01 (the *ruvA* mutant), or the parental clone 5A18NP1. Mice were also inoculated with the previously characterized lp28-1^−^ clone 5A8 [15] as a negative control. Groups of 5 mice were sacrificed at days 14 and 28 post inoculation. Samples of ear, tibiotarsal joint, heart, and bladder were collected for recovery of spirochetes. As expected, the positive control clone 5A18NP1 was fully infectious in both mice strains; spirochetes were isolated from all sites at day 14 and day 28 post inoculation (Table 3). The lp28-1^−^ clone 5A8 exhibited the expected intermediate infectivity phenotype. In normal mice inoculated with 5A8, spirochetes were recovered from only three joint cultures at day 14, and no organisms could be recovered by day 28 post inoculation. However, all tissue sites were infected in C3H/*scid* mice inoculated with 5A8. The *ruvA* mutant exhibited an infectivity phenotype similar to that of lp28-1^−^ *B. burgdorferi* at day 14, but resulted in some positive cultures at day 28 post inoculation (Table 3). In C3H/HeN mice infected with the *ruvA* mutant, only 3 joint cultures of 20 possible cultures were positive for spirochetes at day 14 post inoculation, whereas 20 of 20 cultures were positive in the C3H/*scid* mouse group. At day 28, the *ruvA* mutant was recovered from 2/5 ear, 2/5 tibiotarsal joint, 2/5 heart, and 1/5 bladder cultures in immunocompetent mice, while again all cultures were positive in the C3H/*scid* mouse group. These results indicate that the *ruvA* mutant is significantly impaired in infectivity compared to the parental strain; it is not, however, as severely compromised as the strain lacking lp28-1.

**Table 3:** Infectivity phenotype of the *ruvA* mutant T11P01A01 in immunocompetent C3H/HeN and immunodeficient C3H/*scid* mice is similar to that of the lp28-1^−^ clone 5A8.

		Day14						Day28					
		No. of cultures positive/total					No. of mice positive/total	No. of cultures positive/total					No. of mice positive/total
Mouse strain	*B. burgdorferi* B31 clone	Skin	Joint	Heart	Bladder	All sites		Skin	Joint	Heart	Bladder	All sites	
C3H/HeN	5A18NP1	5/5	5/5	5/5	5/5	20/20	5/5	5/5	5/5	5/5	5/5	20/20	5/5
C3H/HeN	*ruvA* mutant	0/5	3/5	0/5	0/5	3/20	3/5	2/5	2/5	2/5	1/5	7/20	3/5
C3H/HeN	5A8	0/5	3/5	0/5	0/5	3/20	3/5	0/5	0/5	0/5	0/5	0/20	0/5
C3H/*scid*	5A18NP1	5/5	5/5	5/5	5/5	20/20	5/5	5/5	5/5	5/5	5/5	20/20	5/5
C3H/*scid*	*ruvA* mutant	5/5	5/5	5/5	5/5	20/20	5/5	5/5	5/5	5/5	5/5	20/20	5/5
C3H/*scid*	5A8	5/5	5/5	5/5	5/5	20/20	5/5	5/5	5/5	5/5	5/5	20/20	5/5

### RuvAB is not required for survival in ticks

We also tested the ability of *B. burgdorferi* carrying the *ruvA* or *ruvB* mutation to survive in ticks. *I. scapularis* nymphs were inoculated with the *ruvA* mutant T11P01A01, the *ruvB* mutant T03TC051, or the parental strain 5A18NP1 by capillary feeding. Ticks were held for 21–25 days and cultured for *B. burgdorferi* either before or immediately after feeding on 3 C3H/HeN mice. *B. burgdorferi* were readily detected in both fed and unfed ticks, as determined by direct fluorescent antibody (DFA) staining and culture (Table 4). This result indicates that the *ruvA* and *ruvB* mutations do not abrogate the ability of *B. burgdorferi* to survive in ticks. In addition, the mutant spirochetes were able to multiply upon tick feeding (Table 4). Fewer spirochetes were recovered from fed ticks carrying the *ruvB* mutant as compared to the *ruvA* mutant or the parental strain, but enough spirochetes were present in fed ticks to expect a mouse infection to result. However, tissues collected 4 weeks post tick inoculation with the *ruvA* or *ruvB* mutants were consistently culture negative (Table 4). The failure to recover organisms from any cultures derived from tick-inoculated mice (as compared to the sporadically positive cultures from needle-inoculated animals; Table 2) may be due to the lower number of organisms delivered during tick inoculation as compared to needle inoculation, coupled with the decreased survival of *ruvA* or *ruvB* mutants in immunocompetent mice at 28 days post inoculation (Table 2).

**Table 4:** *B. burgdorferi ruvA* and *ruvB* mutants survive well in ticks, but do not establish long-term infection in mice following tick-mediated inoculation.

*B. burgdorferi* Strain	Unfed Ticks			Fed Ticks			Mouse Tissues
	Positive Cultures	Mean no. of spirochetes per field±SD	p value (Mutant vs. WT)	Positive Cultures	Mean no. of spirochetes per field±SD	p value (Mutant vs. WT)	Positive Cultures
*ruvA* mutant	9/10	0.93±0.42	0.77	24/24	27.3±16.2	0.841	0/12
*ruvB* mutant	9/10	1.41±0.44	0.17	24/25	12. 4±7.6	0.053	0/12
5A18NP1	8/10	1.06±0.33		26/26	24.4±24.1		12/12

*I. scapularis* nymphs were inoculated with the *ruvA* mutant T11P01A01, the *ruvB* mutant T03TC051, or the parental strain 5A18NP1 by capillary feeding, as described in Materials and Methods. Ticks were held for 21–25 days and cultured for *B. burgdorferi* either before or immediately after feeding on 3 C3H/HeN mice. The mice used for feeding were euthanized 4 weeks afterwards, and heart, bladder, ear, and tibiotarsal joint tissue specimens were utilized for *B. burgdorferi* culture. Spirochetes were quantified by direct immunofluorescence either in flat or fed ticks. One half of each tick was used for culture and the other half for immunofluorescence. Statistical significance (p<0.05) was assessed by ANOVA.

### Inactivation of *ruvA* or *ruvB* results in reduced *vlsE* recombination

Spirochetes recovered from infected mice were isolated by colony formation, and the *vlsE* cassette region sequences of the resulting clones were determined by PCR amplification and sequencing to examine the effect of *ruvA* or *ruvB* mutation on *vlsE* recombination. For the *ruvA* mutant T11P01A01, a total of 382 *vlsE* sequences were analyzed, comprising sequences from both immunocompetent and SCID mice, at three time points and from four tissues. For the *ruvB* mutant T03TC051, a total of 62 clones isolated from C3H/HeN mice were sequenced, including 22 obtained from skin cultures 7 days post inoculation, and 40 clones from joint, heart, bladder and skin cultures 28 days after inoculation.

**Figure 2:**
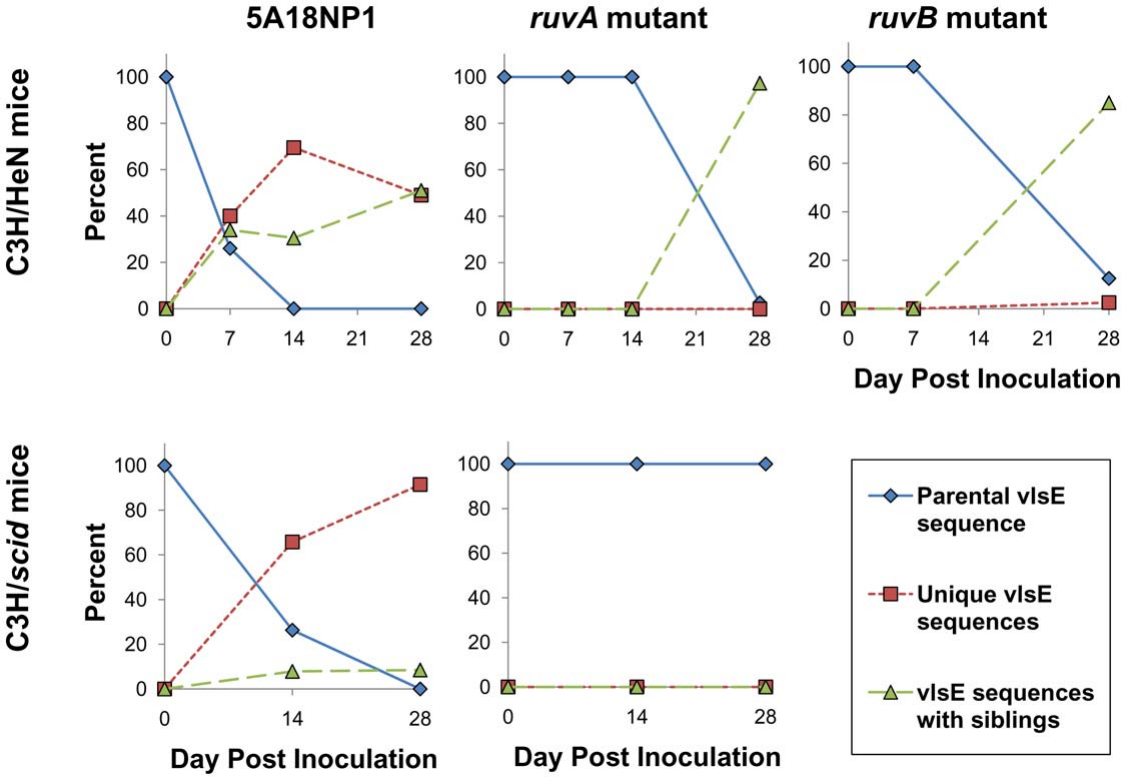
*vlsE* sequence variation observed with the parental strain 5A18NP1 and the *ruvA* mutant T11P01A01 and the *ruvB* mutant T03TC051 following inoculation of immunocompetent C3H/HeN mice and immunodeficient C3H/*scid* mice. The combined results from all tissues examined for each time point are shown. The *vlsE* cassette regions of individual *B. burgdorferi* clones were amplified by PCR, sequenced, and characterized as either identical to the parental *vlsE* sequence, a unique *vlsE* sequence for that mouse, or two or more clones with an identical *vlsE* variant sequence from a mouse (*vlsE* sequences with siblings).

The overall pattern of *vlsE* recombination observed is depicted in Figure 2. For each mouse group and time point, it was determined whether the recovered clones retained the parental *vlsE* sequence, had a unique *vlsE* sequence, or shared the same *vlsE* variant sequence when compared to other clones from the same animal (‘*vlsE* sequences with siblings’). It has been determined previously that *vlsE* variation is not detectable during in vitro culture [22]; therefore all inoculated organisms initially contained the parental *vlsE* sequence.

As observed in prior studies with *B. burgdorferi* B31 clones, the parental strain 5A18NP1 underwent rapid *vlsE* recombination in both C3H/HeN and C3H/*scid* mouse hosts. By 14 days post inoculation, 0/59 clones from C3H/HeN mice had retained the parental sequence. Clones with unique *vlsE* cassette region sequences predominated after 7 days, although a surprisingly high proportion of sibling variants was observed in C3H/HeN mice inoculated with 5A18NP1 (Figure 2, Table S1). At 14 and 28 days post inoculation, no clones with the parental *vlsE* sequence were detected. A similar pattern was observed during infection of C3H/*scid* mice, although the dilution of parental sequence clones occurred at a slower rate; 19/76 clones (25%) still had the parental sequence on day 14. However, even in C3H/*scid* mice no clones with the parental *vlsE* sequence were recovered at day 28 post inoculation.

In contrast, sequence analysis demonstrated that very little *vlsE* sequence variation occurred during infection of mice with the *ruvA* mutant T11P01A11 (Figure 2, Table S1). In immunocompetent mice, only clones with the parental sequence were recovered up to 14 days post inoculation. By day 28 very few (3 of 114, 3%) of the clones retained the parental sequence; however, the remaining clones were quite restricted in terms of sequence variation, with each animal possessing only 1 or 2 variant types. Surprisingly, only parental sequence clones were recovered from C3H/*scid* mice on days 14 and 28 post inoculation, indicating that clones that had not undergone *vlsE* variation predominated in the absence of immune selection. The *ruvB* mutant T03TC051 also exhibited reduced *vlsE* sequence variation during infection of C3H/HeN mice (Figure 2, Table S1).

**Figure 3:**
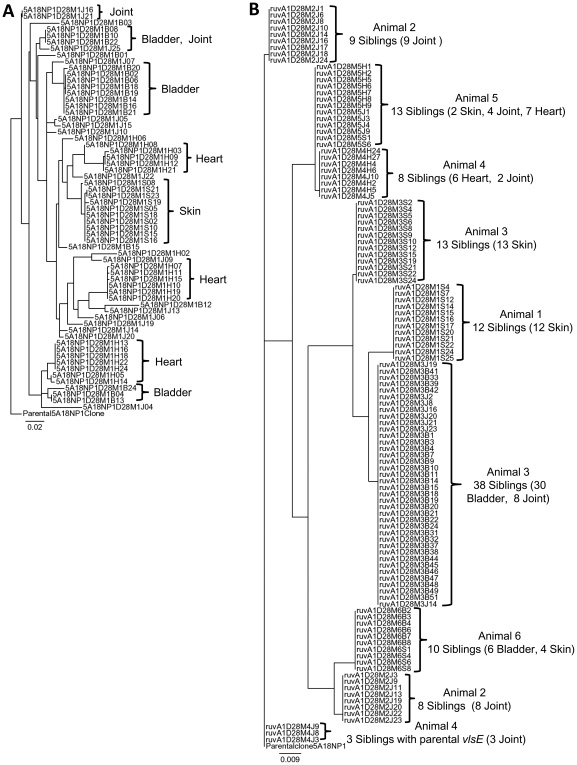
Reduced *vlsE* sequence diversity generated following inoculation of C3H/HeN mice with the *ruvA* mutant T11P01A01. Clones were isolated from C3H/HeN mice 28 days post inoculation with (A) the parental strain 5A18NP1 or (B) T11P01A01. The *vlsE* cassette region sequences of each clone were optimally aligned and then analyzed for sequence diversity using a phylogenetic tree program. The 5A18NP1 clones were isolated from a single mouse, whereas the T11P01A01 clones were obtained from all positive cultures of 6 inoculated mice. The groups of clones isolated from each mouse and their tissue source are indicated. In (A), brackets demarcate clusters of related (but often nonidentical) clones that occurred in the mouse infected with 5A18NP1. In the 5A18NP1 group, there were 31 unique *vlsE* sequences, 10 sequences with siblings, and 0 parental sequences. In (B), brackets indicate clones with identical *vlsE* sequences that were isolated from the 6 mice, frequently from multiple tissues. In this group of clones from mice inoculated with the *ruvA* mutant, there were 0 unique sequences, 8 sequences with many siblings, and 3 parental sequences. Trees are rooted with the 5A18NP1 parental *vlsE* sequence. A larger version of this figure is available at the website http://www.uth.tmc.edu/pathology/borrelia/.

The differences between sequence variation in 5A18NP1 and T11P01A01 are further illustrated in Figure 3. Sequence diversity among the clones isolated at day 28 post inoculation from C3H/HeN mice was analyzed using phylogenetic tree software. The 63 clones from infection with 5A18NP1 were from joint, heart, bladder, and ear tissues of a single mouse, whereas the 114 clones for T11P01A01 were from the 12 culture-positive samples from 6 different mice. With clone 5A18NP1, 31/63 (49%) of the clones characterized possessed unique *vlsE* sequences; the remaining clones were variant siblings that were isolated from the same mouse tissue. Moreover, closely related sequences were often isolated from the same tissue (Figure 3A, brackets), indicating that *vlsE* sequences in sibling clones were diverging within those tissues. For the *ruvA* mutant T11P01A11, only eight different *vlsE* sequences could be detected among the 114 clones examined in the 6 mice, and no more than two different sequences were found in any one animal (Figure 3B). Moreover, clones with the same sequence were often found in more than one tissue site (Figure 3B, brackets). Three clones that retained the parental *vlsE* sequence were also isolated. These results provide further evidence that the *ruvA* mutant is severely compromised in its ability to undergo *vlsE* recombination.

The sequences of 17 bp direct repeats at the 5′ and 3′ ends of *vlsE* central cassette were examined in all of the *vlsE* sequences recovered from *ruvA* mutants. No changes were identified indicating that *vlsE* recombination was not perturbed in the *ruvA* mutants due to instability of the direct repeats during mouse infection and/or *vlsE* recombination.

In *E. coli* and other organisms, *ruvA* and *ruvB* act in concert to promote DNA branch migration. Therefore, we also examined the effects of *ruvB* mutation on *vlsE* sequence variation in C3H/HeN mice. As shown in Figures 2 and S3 and in Table S1, the *ruvB* mutant T03TC051 exhibited reduced *vlsE* recombination rates similar to those observed for the *ruvA* mutant. No *vlsE* variants were observed at 7 days post inoculation. On day 28, the *vlsE* cassette region sequences of 12 to 14 clones from each of three mice were determined. Each animal was found to have only 1 to 3 *vlsE* variant sequences, and 5 clones with the parental *vlsE* sequence were isolated from Animal 3 (Figure S3). Only one clone with a unique *vlsE* variant sequence was identified (1/40, 2.5%). There were subsets of clones with the same *vlsE* variant sequence, consistent with the outgrowth of siblings of rare *vlsE* variants (Figures 2 and S3). Thus the *ruvB* mutant T03TC051 had an infectivity and *vlsE* variation phenotype that was quite similar to that observed for the *ruvA* mutant T11P01A01.

### Analysis of *vlsE* recombination events in the *ruvA* and *ruvB* mutants

**Figure 4:**
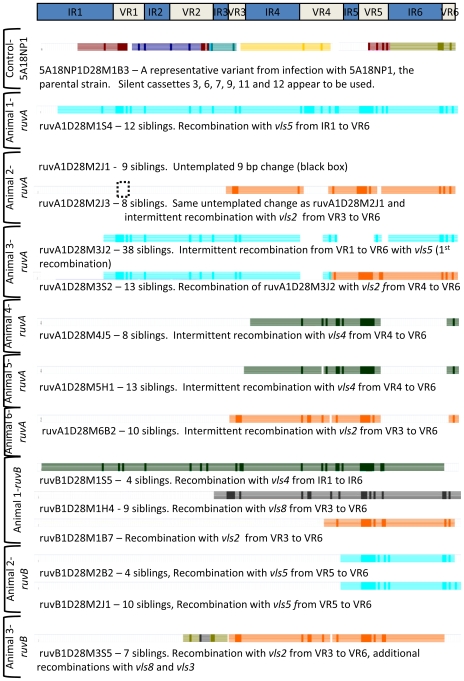
Low complexity of *vlsE* recombination events in *ruvA* and *ruvB* mutants. The positions of the six variable regions (VR1–VR6) and six relatively invariant regions (IR1–IR6) within the *vlsE* central cassette region are provided at the top of the figure. The locations and silent cassette sources of the most likely recombination events for each clone depicted are indicated. A representative clone from a 28 day infection with the parental strain 5A18NP1 is shown at the top. The remainder of the figure shows the *vlsE* variant clonotypes isolated from C3H/HeN mice 28 days post inoculation with the *ruvA* mutant T11P01A01 or the *ruvB* mutant T03TC051. The possible involvement of each of the silent cassettes in sequence variation was analyzed using an Excel®/Visual Basic program, as described previously [11] and depicted in Fig. S2. The horizontal colored bars represent regions of each silent cassette (*vls2* to *vls16*, top to bottom) that match the sequence changes found in the variant clone. Dark regions in each bar correspond to the actual sequence changes, whereas the lighter portion of the bar represents the maximum possible region of that silent cassette exchanged into *vlsE* to produce the observed sequence change. In isolates from Animal 2 inoculated with the *ruvA* mutant, a 9 bp untemplated change (black hatched box) that did not match the *vls* silent cassettes or any other genomic sequence was present in all clones isolated.

The non-parental *vlsE* variants recovered 28 days post infection with the *ruvA* and *ruvB* mutants were analyzed to determine the length, location and silent cassette source of the recombination events (Figure 4, Figure S2). Each sequence was aligned with parental *vlsE* sequence, codon formatted and analyzed using a semi-automated analysis program [11]. The program compares each codon to parental and silent *vls* sequences and depicts the result (identity or no identity) as colored bars with each silent cassette assigned a unique color. Dark regions represent the sequence changes that may have resulted by recombination with that silent cassette. The lighter colored regions are contiguous codons that match both parental and silent cassette sequences and thus represent the possible boundaries of a recombination event.

The most likely recombination events are presented in Figure 4; a more detailed presentation showing all silent cassettes potentially involved in the recombination events is shown in Figure S2. Variants recovered from infections with 5A18NP1 at day 28 of infection are usually difficult to assign to a single silent cassette with confidence and appear to have undergone multiple recombination events ([4] and data not shown). For example, the simplest explanation for the variant recovered from a mouse infected with 5A18NP1 (Figure 4, first panel) appears to involve multiple recombinations of varied lengths with 6 different silent cassettes. In contrast, the results obtained with the *ruvA* and *ruvB* mutants were consistent with the occurrence of relatively rare and elongated *vlsE* recombination events (Figure 4). This pattern is exemplified by the *vlsE* sequence recovered from animal 1, which likely represents a single recombination event with *vls5* spanning almost the entire cassette region. Although many silent cassettes have sequence identity to the variant residues, *vls5* represents the only continuous match over the entire region (Figure S2). One variant (animal 2) is predicted to have undergone untemplated changes (not matching any *vlsE* silent cassette) in VR-I followed by an intermittent recombination event with *vls2*. Several of the recombination events found in the *ruvA* mutant appear to be intermittent, i.e. the recombination events were discontinuous over the length of the cassette, alternating between the template cassette and *vlsE1*. This intermittent recombination has been previously observed in *vlsE* recombination [11]. In addition, we noted that all recombination events from *ruvA* mutants included variable regions IV through VI. This bias is not observed with the parental strain [4]. Equally striking is length of the recombination events observed in the *ruvA* mutant. Coutte et al. [11] analyzed 126 clones that had undergone a single recombination event and identified the minimum and maximum predicted lengths of recombination. The majority of these clones (55%) had minimum recombination lengths of 1–5 nucleotides and only 28% had minimum recombination lengths of >15 nucleotides. The variants obtained from *ruvA* mutant infections, however, had a median minimum recombination length of 168 bp (range 91 to 479 bp), and a median maximum recombination length of 269 bp (range 111 to 546 bp).

Another unusual feature of the recombination events seen in the *ruvA* mutant is the distribution of silent cassette usage. Only 3 silent cassettes, *vls2*, *vls4*, and *vls5* were used in the seven templated variants. *vls4* and *vls5* were each used twice. *vls2*, the silent gene most proximal to the expression cassette has been observed to be used very rarely in infections with wild type *B. burgdorferi*, yet we observed it in three of seven templated *ruvA* variants. These results suggest that the normal mechanism of silent template selection may be perturbed in *ruvA* mutants.

The *vlsE* sequences derived from infection with the *ruvB* mutant displayed similar features to those from the *ruvA* mutant (Figures 4 and S2). Most sequences were the result of single recombination events and used few *vls* silent cassette templates. The recombinations observed were unusually long, with a median minimum recombination length of 151 bp (range 119 to 389 bp) and an average maximum length of 179 bp (range 151 to 561 bp). Recombination events always included most of the 3′ end of the recombination cassette; however, these recombinations did not always include VR6, as observed for the recombination events derived from *ruvA* mutants.

### Antibody responses to *vlsE* in *ruvA* and *ruvB* mutants

To determine whether mice infected with the *ruvA* and *ruvB* mutants developed a robust antibody response to VlsE, we performed ELISAs on serum from infected animals. We found that serum from animals infected with the *ruvA* or *ruvB* mutants displayed more variable antibody responses to VlsE than animals infected with the parental *B. burgdorferi* strain (data not shown). Some animals displayed similar reactivity to sera from animals infected with parental strain while others displayed lower anti-VlsE activity. However, low anti-VlsE titers did not necessarily correlate with positive *B. burgdorferi* cultures from harvested organs. It is speculated that low quantities of anti-VlsE antibodies in some mice may correspond to early clearance of *B. burgdorferi*, thus resulting in a limited stimulation of the immune response against *Borrelia* antigens, including VlsE.

### Attempted complementation of the *ruvA* mutant

In an attempt to fully demonstrate the role of *ruvA* in *vlsE* sequence variation and infectivity, we complemented the *ruvA* mutant T11P01A01 with the shuttle vector pKFSS1 [35] containing either *ruvA* alone, *ruvAruvB*, or *ruvAruvBqueApfpB* (the four genes in the predicted operon; Figure S1). A 264-bp region upstream of *ruvA* predicted to include the transcriptional promoter was included in all three constructs. The integrity of the resulting plasmids was confirmed by sequencing the inserts, and transformation of T11P01A01 was demonstrated by streptomycin selection followed by PCR using one primer specific for the pKFSS1 vector sequence and one primer corresponding to the *B. burgdorferi* DNA insert. Groups of three mice were each inoculated intradermally (10^5^ organisms/mouse) with one of two clones containing each complementation construct, yielding 3×6 = 18 mice inoculated with complemented lones. The two control groups (3 mice each) were inoculated with positive control 5A18NP1 and the non-complemented *ruvA* mutant T11P01A01. All strains yielded positive cultures from each of 3 skin sites taken distant from the inoculation site on day 7, indicating that the infection was successful in each case. Six representative day 7 cultures were tested for the presence of the complementing plasmids by PCR, and yielded the expected amplicons. All cultures except those from 5A18NP1-inoculated mice were negative on day 14 post inoculation, and on day 28 the proportions of positive clones for the complemented clones were similar to that obtained for the *ruvA* mutant T11P01A01 (data not shown). In addition, 17 complemented clones isolated on day 7 post inoculation were examined for *vlsE* recombination, and none exhibited *vlsE* sequence changes; this result was similar to what had been observed previously with uncomplemented *ruvA* mutant clones at this time point (Figure 2, Table S1). Therefore attempts to restore infectivity and *vlsE* sequence variation to wild type levels by complementation have thus far been unsuccessful.

### Inactivation of *ruvA* does not result in increased sensitivity to DNA damage

**Figure 5:**
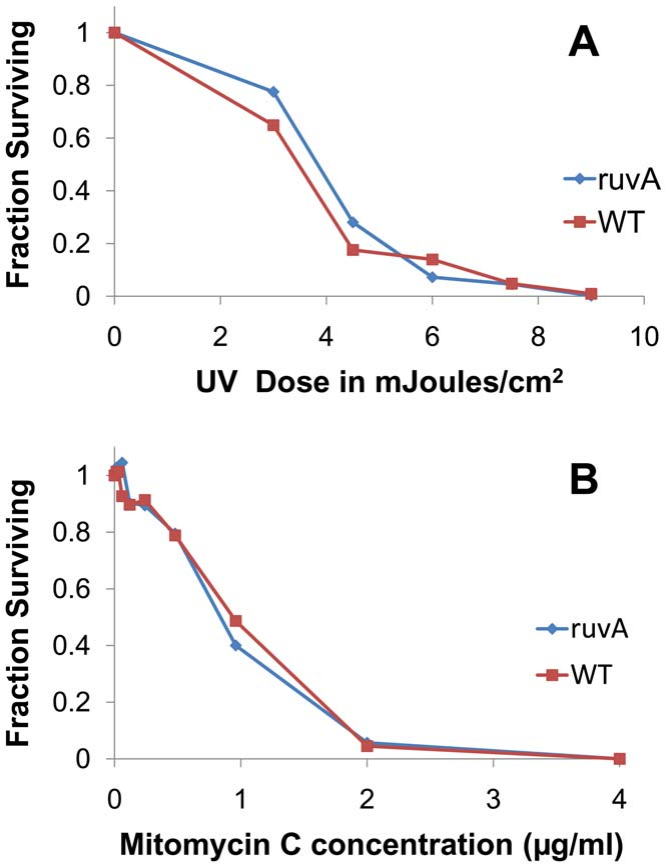
The *ruvA* mutant T11P01A01 does not exhibit enhanced sensitivity to UV light or mitomycin C. (A) Survival of *ruvA* mutant and parental strain 5A18NP1 after exposure of UV light. The dosages of UV irradiation (254 nm) applied to the bacterial suspensions are indicated. (B) Survival of *ruvA* mutant and parental strain 5A18NP1 after 14 h treatment with the indicated doses of mitomycin C in BSK II medium. Colony counts on duplicate or triplicate plates were utilized to determine the concentration of surviving *B. burgdorferi*. Results shown are for representative experiments from three independent studies for each treatment.

In many microorganisms, *ruvA* is important in the repair of DNA damage resulting from UV irradiation or chemical mutagens. To assess the role of *B. burgdorferi ruvA* in DNA repair we monitored the survival of the *ruvA* mutant T11P01A01 subjected to DNA damaging conditions. Log phase cultures were irradiated with increasing doses of UV (254nm) light, and cultured to determine spirochete survival by colony counting. Figure 5A shows the results of a representative experiment. The parental strain 5A18NP1, and *ruvA* mutants had a similar number of colonies surviving at all UV doses, indicating that *ruvA* mutants do not have increased sensitivity to UV irradiation.

Mitomycin C causes DNA damage by crosslinking complementary DNA strands at CpG sequences and is a commonly used reagent to examine the cellular response to DNA damage. To determine the sensitivity of the *ruvA* mutants to mitomycin C we assessed the viability of the *ruvA* mutant T11P01A11 and the parental strain 5A18NP1 after 14 hours exposure to increasing doses of mitomycin C. Treated cultures were plated and the number of colonies visible after 2–3 weeks incubation was used to assess spirochete survival. Figure 5B shows the survival of *B. burgdorferi* colonies after exposure to varied concentrations of mitomycin C. We observed no differences in survival between the *ruvA* mutant and the parental strain 5A18NP1.

## Discussion

We conducted a genetic screen for trans-acting factors involved in *vlsE* antigenic variation in *B. burgdorferi*. Of the seven genes examined in this screen, four exhibited diverse PCR-RFLP patterns of *vlsE*, indicating that the inactivated genes are not centrally involved in *vlsE* recombination in C3H/HeN mice. Mutant MG065 in BB0098 (annotated as a recombination and DNA strand exchange inhibitor and as *mutS-II* in other organisms) was not recovered from inoculated mice, and thus could not be evaluated for *vlsE* variation. Two of the remaining transposon mutants, which had disruptions in the *B. burgdorferi* orthologs of the Holliday junction helicase genes *ruvA* and *ruvB*, had reduced *vlsE* recombination. The only other mutant that exhibited reduced infectivity and PCR-RFLP diversity was a *mutS* mutant, T05P01C02. A second *mutS* mutant, T10P01D06, had an insertion site near the end of the gene (insertion ratio 0.97); this clone had normal infectivity and RFLP results, indicating that a functional product is likely produced. The possible role of *mutS* in *vlsE* recombination will be investigated further in a separate study.

The predicted proteins encoded by the *B. burgdorferi* BB0023 and BB0022 open reading frames have a high degree of homology with the Holliday junction helicase proteins RuvA and RuvB, respectively, of other organisms. In *Escherichia coli*, the 22-kDa protein RuvA specifically targets RuvB to the Holliday junctions [36],[37], and the combined action of RuvAB results in branch migration during homologous recombination [38]. The 19-kDa RuvC resolvase cleaves the Holliday junction and is required for resolution of Holliday junctions in *E. coli* [39]. No homolog of RuvC has been identified in Lyme disease *Borrelia*, although RuvC orthologs have been identified in other spirochetes including the relapsing fever organism *B. turicatae*, *T. pallidum*, *T. denticola* and *L. interrogans*. Aravind et al. has proposed that a family of predicted LE exonucleases may substitute for RuvC in *B. burgdorferi* [40]. In addition to their role in recombination, *E.coli ruv* mutants exhibit defects in DNA repair such as increased sensitivity to UV light and mitomycin C [41],[42].

The infectivity phenotypes of the *ruvA* mutant T11P01A01 and the *ruvB* mutant T03TC051 resemble those of *B. burgdorferi* strains that lack lp28-1 [14],[15],[17],[18] or the *vls* locus [19], although some differences exist. As shown in Tables 2 and 3, the *ruvA* and *ruvB* mutants exhibited lower culture positivity than the parental strain 5A18NP1 in immunocompetent C3H/HeN mice. The *ruvA* mutant culture positivity pattern was the same as the lp28-1^−^ clone 5A8 on day 14 post inoculation in C3H/HeN mice, with only joint specimens yielding positive cultures (Table 3); however, 7/20 cultures in diverse tissues were positive for the *ruvA* mutant at day 28 post inoculation, as compared to a lack of positive cultures in the 5A8-inoculated animals. The ability of some *ruvA^−^* organisms to survive is most likely due to the ability to express VlsE and to undergo limited *vlsE* recombination. The *ruvA* mutant and the lp28-1^−^ clone 5A8 is able to infect all tissues in C3H/*scid* mice, as has been observed previously with lp28-1^−^ strains and clones in which the *vls* locus was deleted from lp28-1 by telomere-mediated plasmid truncation [19]. Thus mutation of *ruvA* results in reduced ability to survive in the presence of the adaptive immune system, perhaps due to antibody responses against invariant or limited variation forms of VlsE.

We also tested the ability of *B. burgdorferi* carrying *ruvA* or *ruvB* mutations to survive in ticks. *B. burgdorferi* were readily recovered from both fed and unfed ticks suggesting that the ruvA and ruvB mutations do not abrogate the ability of *B. burgdorferi* to survive in ticks. However, the mutant spirochetes did not establish infection in C3H/HeN mice following tick inoculation (Table 4). These data suggest that the lack of detectable organisms at day 28 post mouse inoculation (Table 4) was due to the effects of *ruvA* and *ruvB* mutations on survival in mice rather than poor replication in ticks or transmission from ticks to mammals. The failure to recover organisms in cultures derived from mouse inoculation via ticks may be due to the relatively low number of organisms delivered during tick inoculation as compared to the 10^5^ *Borrelia* per mouse used in the needle inoculation studies.

The reduced ability of the *ruvA* mutant T11P01A01 to undergo *vlsE* recombination is illustrated in Figures 3, 4, and S3. Figure 3 shows that *vlsE* variants were not detected on days 7 and 14 in C3H/HeN mice inoculated with the *ruvA* mutant; while *vlsE* variants are predominant on day 28, only one or two variant clones are detected in each mouse (Figures 4 and 5). Remarkably, no *vlsE* variants were detected at any time point following infection of C3H/*scid* mice with the *ruvA* mutant, whereas all of the clones examined at 28 days post inoculation were variants when C3H/*scid* mice were inoculated with the parental 5A18NP1 strain. Combining the reduced culture positivity results with the decreased *vlsE* variant diversity observed with the *ruvA* mutant, the following is evident: 1) inactivation of *ruvA* results in reduction of *vlsE* recombination rates, resulting in only a few variants in C3H/HeN mice and the lack of detected variants in C3H/*scid* mice; and 2) in C3H/HeN mice, the few clones that have undergone *vlsE* recombination are able to survive after the anti-VlsE response is activated, whereas nearly all of the parental clones are eliminated by day 28 post inoculation.

The types of recombination events observed in the *ruvA* mutant were also quite different than those observed in wild-type *B. burgdorferi*. The average length of observed recombination events was an order of magnitude larger than in wild type clones. Moreover, most of the predicted recombination events observed in the *ruvA* mutant also appeared to represent intermittent recombination events, with regions of recombination interrupted by stretches of the unchanged, parental *vlsE* sequence (Figure 5). Finally, *vls2*, *vls4*, and *vls5* were the only silent cassettes utilized in the *ruvA* mutant. The *ruvB* mutant also exhibited a limited repertoire of silent cassette usage (cassettes 2, 3, 4, 5, and 8). Taken together, these results may indicate that the recombination process is different in the *ruvA* mutant than in wild-type organisms; i.e., the observed changes could involve a different, underlying recombination mechanism other than the as yet cryptic, *vlsE*-specific process that appears to be induced during mammalian infection.

We found that sensitivity of *B. burgdorferi* to UV radiation and mitomycin C exposure was not affected by *ruvA* mutation. In other bacteria such as *E. coli*, deficiencies in RuvA, RuvB, or RuvC result in increased sensitivity to DNA-damaging agents, including UV irradiation and mitomycin C [43]. UV irradiation causes intra-strand thymine dimers in DNA, and the damage is repaired by RecA-mediated SOS response [44]. Mitomycin C exposure induces inter-strand cross-link in DNA, and an extended SOS response is needed to repair the damaged DNA [45]. Induction of genes encoding DNA repair enzymes during the SOS response is one of the most important DNA repair system in bacteria [46]. However, the *B. burgdorferi* genome does not encode a homolog of the SOS response repressor LexA, and SOS boxes (LexA binding sites in promoter regions) are not present upstream of *recA* and other genes that comprise the SOS regulon [47]. Also, expression of *recA* is not induced following UV exposure [48]. Thus *B. burgdorferi* appears to lack an SOS regulon. As an obligate parasite, *B. burgdorferi* is never exposed directly to UV light, and thus may have lost its ability to upregulate genes involved in the repair of DNA damage caused by radiation and other agents.

Thus far, our attempts to restore full infectivity and *vlsE* recombination through complementation with *ruvA* in the shuttle vector pKFSS1 have been unsuccessful. In addition, we have also tried using constructs containing *ruvAruvB* and *ruvAruvBqueApfpB*, in case the transposon insertion in *ruvA* has some polar effect on the expression of downstream genes. A 264-bp region upstream of *ruvA* was used in all of these constructs; it is presumed to contain the promoter, although the region lacks a strong consensus promoter sequence (e.g. −35 and −10 sequences with the proper spacing). BB0024, the gene upstream of *ruvA*, is oriented in the opposite direction, indicating that *ruvA* is the first gene in the operon. Dresser et al. [49] have also been unsuccessful in *trans* complementing *ruvA* and *ruvB* mutants obtained by allelic exchange. The *ruvA* and *ruvB* mutants obtained by our laboratory and by Dresser et al. [49] were derived independently by different means (transposon mutagenesis and gene disruption by allelic exchange), yet have nearly identical phenotypes. Thus it is likely that the lack of complementation observed in these studies is due to technical complications, as has been commonly observed in genetic studies involving infectious *B. burgdorferi*. We will continue our efforts to complement the *ruvA* and *ruvB* mutants in an attempt to fulfill the ‘molecular Koch's postulates’ on the roles of these genes in infectivity in immunocompetent mice and in *vlsE* recombination.

The current study along with those by Liveris et al. [31] and Dresser et al. [32] provide some insight into the mechanism of recombination in the *vls* antigenic variation system. The central role of RuvAB indicates that heteroduplex formation with branch migration is important in this process. The lack of a requirement for RecA [31],[32] is surprising. RecA provides two important functions in other microorganisms: ATP-dependent formation of RecA-single-stranded DNA nucleoprotein filaments that facilitate the homologous base pairing during heteroduplex formation [50]; and cleavage of LexA to activate the SOS response through its activity as a co-protease when bound to single-stranded DNA. As indicated above, *B. burgdorferi* lacks a recognizable LexA ortholog, so the latter function is likely to be unimportant (although co-protease activity is present in *B. burgdorferi* RecA). Liveris et al. [31] state that the apparent lack of effect of *recA* mutation on *vlsE* recombination indicates that homologous recombination mechanisms are not involved in this process. However, ‘templated’ *vlsE* sequence changes (those involving incorporation of silent cassette sequences) consistently occur at the same position as the optimal alignment between the silent cassette and *vlsE*; i.e., the observed *vls* silent cassette/*vlsE* recombinations are always homologous, and never nonhomologous [11]. *vlsE* recombination is induced during mammalian infection; it is possible that factor(s) that bind specifically to *vls* sequences and promote strand invasion and heteroduplex formation may be expressed under these conditions and hence fulfill a RecA-like (but site-specific) role. Such factor(s) have not been identified to date. Once a heteroduplex is formed, RuvAB may promote the migration of the heteroduplex branch point, thus extending the region of strand exchange. In a set of 126 *vlsE* variants that appeared to contain a single recombination event [11], the putative minimal recombination events ranged from 1 nt to 349 nt, with a median value of less than 5 nt. This result indicates that the strand replacement process is often interrupted quite soon after its initiation. The observed range of sequence identity between donor and recipient sequences flanking the region of sequence change was from 0 nt to 88 nt, with (remarkably) as little as 6 nt on one side or the other [11]. It is currently unknown whether the role of the RuvAB migrase is to promote heteroduplex formation in these regions of sequence identity (thus nucleating the strand exchange event), or whether it is also able to ‘drive’ the strand pairing through regions of sequence differences.

Gene conversion has been described as the predominant mechanism in other bacterial and protozoal antigenic variation systems, including those of relapsing fever *Borrelia* (e.g. *B. hermsii*), *Neisseria gonorrhoeae*, *Anaplasma marginale*, *Trypanosoma cruzi*, and *Babesia bovis* [51],[52],[53],[54]. Lyme disease and relapsing fever *Borrelia* are closely related, and VlsE and the variable large protein (Vlp) antigenic variation proteins have some sequence similarity. However, in *B. hermsii* the gene conversion events result from homologous recombination within well-demarcated upstream homology sequences (UHS) and downstream homology sequences (DHS), resulting in replacement of nearly the entire expression site gene with a silent gene sequence [53],[55]; therefore it is different from the *vls* system both in terms of mechanism and gene conversion outcome. The *N. gonorrhoeae pilE* system, the *A. marginale MSP2*, and the *B. bovis* VESA1a systems each involve segmental gene conversion events utilizing multiple silent gene copies to produce highly variable pilin or surface protein sequences. The *pilE* system is dependent upon RecA and other recombination system proteins that are not required for *vlsE* recombination, as described in detail by Dresser et al. [32]. In *A. marginale*, the gene conversion event is almost always ‘anchored’ in a conserved region flanking the hypervariable region [56]. The mechanisms of *B. bovis* VESA1a recombination are not well characterized, but are likely to involve eukaryote-specific elements that will differ considerably from those present in the prokaryote *B. burgdorferi*. Thus, based on current knowledge, the *vls* system may represent a unique gene conversion process that is mechanistically dissimilar to other known antigenic variation systems.

## Materials and Methods

### Ethics statement

All procedures involving mice conducted at the University of Texas Health Science Center at Houston were reviewed and approved by the Animal Welfare Committee of that institution. All mouse studies conducted at the Tulane National Primate Research Center were reviewed and approved by its Institutional Animal Care and Use Committee (IACUC).

### Bacterial strains and growth medium

The transformable, infectious *Borrelia burgdorferi* B31 clone 5A18NP1 was used for generation of all mutants. 5A18NP1 is a genetically modified clone in which plasmids lp28-4 and lp56 are missing and BBE02, encoding a putative restriction-modification enzyme, has been disrupted [33]. *Borrelia burgdorferi* B31 clone 5A8, containing all plasmids except lp28-1, was isolated previously from the low-passage strain B31 [15],[57] and was used as negative control in mouse inoculation experiments. All strains used in this study had undergone no more than two subcultures since clone isolation prior to infectivity studies. *B. burgdorferi* were grown at 34°C in 3% CO_2_ in Barbour-Stoenner-Kelly II (BSK-II) medium supplemented with appropriate antibiotics as described previously [58]. The in vitro growth rates of the *ruvA* and *ruvB* mutants and the parental strain 5A18 NP1 were determined by daily quantitation of organisms in triplicate cultures by dark-field microscopy. *E. coli* TOP10, a DH5α™-derived strain obtained from Invitrogen Corporation (Carlsbad, CA, USA), was used for the preparation of plasmids for electroporation into *B. burgdorferi*.

### Inactivation of DNA recombination and repair genes

Genes putatively involved DNA recombination and repair were inactivated by transposon mediated mutagenesis as part of a transposon signature tagged mutagenesis (STM) study on-going in the laboratory. This study will be described in detail in another publication (Lin, T., L. Gao, C. Zhang, E. Odeh, and Norris, S.J., manuscript in preparation). Briefly, twelve independent mutant libraries, each having a unique 7 bp sequence tag, were created using modified versions of the suicide plasmid pMarGentKan. This plasmid was graciously provided by Dr. P. E. Stewart (Rocky Mountain Laboratories, National Institutes of Health, Hamilton, MN) and is a modified version of pMarGent [59] in which a kanamycin resistance cassette (*flaB::aph1*) was inserted in the ‘backbone’ of the vector, outside the *himar1*-based transposable element and a *flgB:aacC1* gene is inserted within the transposable element (P. E. Stewart, unpublished data). After insertion of unique sequence tags into pMarGentKan, 5 µg of each plasmid was electroporated into *B. burgdorferi* B31 using a modification of previously described methods [59],[60]. The transformants were incubated in BSK-II medium without antibiotics for recovery overnight hours and plated in solid BSK-II media with 200 µg/ml of kanamycin and 40 µg/ml of gentamicin as described previously [57]. Colonies were selected and cultured in liquid BSK-II medium until mid-log phase prior to addition of 15% (v/v) glycerol and storage at −70°C. The transposon insertion site was determined by restriction digestion of the *Borrelia* genomic DNA, plasmid rescue in *E. coli*, and sequencing as described previously [59]. Properties of the transposon mutants selected for this study are shown in Table 1.

### Determination of plasmid content

The plasmid profiles of DNA recombination and repair mutants were determined by a microtiter plate-based PCR method as previously described [15] or by a multiplex PCR scheme followed by detection using Luminex® FlexMAP™ technology. The Luminex® procedure will be described in detail in another article (S. J. Norris, J. K. Howell, E. Odeh, T. Lin, and D. G. Edmondson, manuscript in preparation). Briefly, multiplex PCR reactions were performed to amplify plasmid-specific regions. The resultant PCR reactions were treated with exonuclease I and shrimp alkaline phosphatase (Exo/SAP, U. S. Biologicals) to remove excess nucleotides and primers, and then subjected to **a**llele **s**pecific **p**rimer **e**xtension (ASPE) in the presence of biotin-dCTP using primers specific for individual plasmid products. The 5′ end of the ASPE primers contains an xTAG® universal tag sequence and the 3′ end of the ASPE primers contains the plasmid-specific sequences. The 5′ universal tag sequence was hybridized to the complementary anti-tag sequence coupled to a particular xMAP® bead set and biotin labeled DNA was detected with PhycoLink® Streptavidin-R-Phycoerythrin (SAPE). Detection and analysis was carried out using the Luminex® 200™ System (Luminex Corporation, Austin, TX), and samples were scored as plasmid positive or negative based on the median fluorescence intensity values for each probe set.

### Mouse infection studies

Clones with transposon insertions in DNA recombination and repair genes were tested individually for infectivity in 4-week-old C3H/HeNHsd (wild type) by needle inoculation (Table 1). Mutant clones, the parental strain 5A18NP1, and clone 5A8 were cultured to mid-log phase, and groups of 4–5 mice were inoculated with 1×10^5^ organisms subcutaneously at the base of the tail as described previously [57]. Skin biopsies were collected aseptically at day 4, 7, 14 post inoculation. The mice were sacrificed at day 28 post inoculation, and skin, tibiotarsal joint, heart, and urinary bladder were collected. The tissue specimens were cultured in 6 ml BSK II medium containing kanamycin and gentamicin at 34°C in 3% CO_2_. The cultures were checked for spirochetes by dark-field microscopy at 2 and 4 weeks, and positive cultures were diluted and subsurface plated in 0.7% solid BSKII –agarose medium to isolate individual colonies. For selected mutants, similar studies were performed in 5-week-old, female C3H.C-Prkdĉscid/ICRSmnHsd SCID mice (Harlan, Indianapolis, IN). For clarity, this mouse strain is referred to as C3H/*scid* in this article. Anti-VlsE responses were assessed by ELISA as described previously [61].

### Tick inoculation studies

*B. burgdorferi* mutant and wild-type strains were grown in BSK-II medium that was supplemented with 6% rabbit serum (Pel-Freez Biologicals) and 45.4 µg/ml rifampicin, 193 µg/ml phosphomycin, 0.25 µg/ml amphotericin, 200 µg/ml kanamycin (all from Sigma-Aldrich). Gentamicin (Gibco) at 50 µg/ml was added to the mutant strain cultures only. *Ixodes scapularis* nymphal ticks from the Tulane National Primate Research Center tick colony were capillary fed with culture medium that contained the *ruvA* mutant, *ruvB* mutant, or the 5A18NP1 wild type *B. burgdorferi*, each at a concentration of about 9×10^7^ cells/ml. Capillary feeding was performed by a procedure described previously [20]. Ticks were then allowed to rest for 21 to 25 days in a humidified environment at 22°C. Ten flat (unfed) nymphs from each group were surface disinfected and crushed in 30 µl of sterile PBS. Half of this volume was cultured in 5 ml of BSK II medium, supplemented with antibiotics as above, for a total of 12 weeks, and monitored weekly after the second week. . The remaining half was distributed on microscope slides for quantitation of spirochetes (see below).

The remaining flat ticks were placed on mice. Three 8–10 week-old C3H/HeN female mice were used for each *B. burgdorferi* strain, and each mouse received 10–12 nymphs. Engorged (fed) ticks (about 75% of the initial number) were collected within 6 days, and cleaned, crushed, and cultured or prepared for DFA as described above. All of the mice were euthanized 4 weeks after the ticks had dropped off, and heart, bladder, one ear, and one tibiotarsal joint were collected from each animal. In each case, half of the organ sample was snap frozen in liquid nitrogen and stored and the other half was placed in culture for 8 weeks.

### Immunofluorescence

A direct fluorescent antibody (DFA) assay was performed to evaluate survival of each *B. burgdorferi* strain in ticks as described previously [62]. Briefly, tick smears from flat and fed ticks (see above) were air-dried on glass slides, acetone fixed and stored at −20°C until DFA examination. Slides were incubated with 40 µl of a 1∶10 dilution of fluorescein-labeled anti-*Borrelia* species antibody (Kirkegaard & Perry Laboratories (Gaithersburg, MD)) for 30 minutes at 37°C. Following incubation, the slides were washed in phosphate-buffered saline and examined by fluorescence microscopy (magnification, 600X). For unfed ticks and for each spirochetal strain, spirochetes in 10 microscope fields were counted in each of 10 ticks per strain. For fed ticks, between 5 and 10 fields were counted in 24–26 ticks per strain. The mean number of spirochetes per field and the standard deviations are presented in Table 4. Statistical significance (p<0.05) was assessed by ANOVA.

### RFLP analysis and sequencing of amplified *vlsE* cassette regions

As a screen for *vlsE* sequence variation, cultures obtained from mouse infections were subjected to *vlsE* cassette region amplification followed by RFLP analysis. Five µl of unpurified culture was used as the DNA template. The *vlsE* expression cassette was amplified by using primers 4066 and 4120 [6] and the Phusion™ High-Fidelity DNA Polymerase (Finnzymes, Inc. Woburn, Massachusetts, USA). PCR reactions were performed in volumes of 50 µl containing 10 µl of 5× HF buffer with 7.5 mM MgCl_2_, 1 µl of 10 mM dNTPs, 1 µl of 25 mM primers, and 1 U of Phusion™ High-Fidelity DNA Polymerase. PCR reactions were performed in a Eppendorf Mastercycler® thermocycler (Foster City, California, USA), using the following conditions; 98°C for 2 min followed by 30 cycles of 1) denaturation at 98°C for 10 sec, 2) annealing at 61°C for 20 sec, and 3) extension at 72°C for 45 sec and a final extension at 72°C for 10 min. For RFLP analysis, 10 µl of PCR product was digested with 2.5 U of the restriction endonuclease *HphI* (New England BioLabs, Ipswich, MA) for 2 hrs at 37°C in 10×NEBuffer 4. The digests were separated in a 2% (w/v) agarose gel at a 100V constant voltage for 1.5 hrs in the presence of ethidium bromide. The Hi-Lo™ DNA marker (Bionexus, Oakland, CA) was used as a molecular size marker. Gels were imaged with UV light illumination.

For sequence determination, the PCR products from individual clones were amplified as described above, purified using the Qiaquick® PCR purification kit (Qiagen), and sequenced on both strands at the High-Throughput Genomics Unit (Department of Genome Sciences, University of Washington, Seattle), using the same primers used for PCR amplification. Each DNA sequence was compared with their corresponding chromatographs and the parental *vlsE* sequence to verify the quality and accuracy of the sequence data.

### *vlsE* sequence analysis

The sequence of the *vlsE* cassette region of clone 5A18NP1 (GenBank GQ369288) is identical to that of *B. burgdorferi* B31-5A3, from which the *vlsE* sequence (U76405) was derived initially [6]. All *B. burgdorferi* clones and the GenBank numbers corresponding to their *vlsE* cassette region sequences are listed in Table S3. For simplicity, the clone names refer to all clones resulting from T11P01A01 infection as ruvA1 and all T03TC051 derivatives as ruvB1. All clones resulting from infection with the parental clone are given the prefix 5A18NP1. The clone names (e.g. RuvA1SD14M1S2) are in the following format: infecting strain name; S indicates C3H/*scid* mice (all others are C3H/HeN mice); D7, D14 or D28 = 7, 14 or 28 days post inoculation; mouse number (M1, M2, etc.); S, E, J, H, B = skin, ear, tibiotarsal joint, heart, and bladder, respectively; and the clone number from that tissue. The clone numbers and their GenBank accession numbers are also provided at the website http://www.uth.tmc.edu/pathology/borrelia/.

*vlsE* sequence analysis of variants was performed as described previously [11]. The output of this Excel®/Visual Basic-based program is a color-coded map of possible *vlsE* recombination events, as shown in Figure 4 and Figure S2. The minimum and maximum deduced regions of recombination were determined by analyzing the longest contiguous recombination event in the variant sequence. The relationships among clones with different *vlsE* sequences were displayed graphically as phylogenetic trees. Phylogenetic trees were constructed using the online phylogenetic program Phylogeny.fr [63], based on the aligned parental and variant *vlsE* sequences.

### Sensitivity to UV radiation and mitomycin C

The *ruvA* mutant T11P01A01 and the parental strain 5A18NP1 were tested for UV sensitivity using a modification of the method described by Miller, *et al*. Cultures were grown to a density of 1×10^7^ organisms/ml. The spirochetes were centrifuged and resuspended in phosphate buffered saline (PBS) at a concentration of 1×10^8^ organisms/ml, and 0.1 ml of the suspensions was rapidly aliquoted into 24-well plates. The bacteria were irradiated with UV light (254 nm) using a Spectrolinker XL-1000 UV cross-linker (Spectronics, Westbury, N.Y.) at doses of 0, 3.0, 4.5, 6.0, 7.5 and 9.0 mJ/cm^2^. The UV treated bacteria were protected from room light, diluted in BSK-II and cultured in BSK-II plates in duplicate or triplicate, at a concentration of 100 organisms per plate. The colonies were counted after 2 weeks incubation.

For determination of mitomycin C sensitivity, fresh cultures of *Borrelia* were grown to mid-log phase and diluted to a density of 1×10^7^ organisms/ml. Mitomycin C (Sigma-Aldrich, St. Louis, MO) was added to 1 ml cultures at final concentrations of 0, 15, 30, 60, 120, 240, 480, 960, 2000, and 4000 ng/ml, and the cultures were incubated at 34°C in 3% CO_2_. The concentration of motile spirochetes was determined by dark-field microscopy at 2, 4, 6, 8, 12, 16, and 24 hours. At 14 hours, the cultures were serially diluted in BSK-II to obtain concentrations 1000 cells/ml, and 0.1 ml portions of each dilution were cultured in BSKII plates with antibiotics as described above. Each culture was plated in duplicate and incubated 7–10 days at 34°C. Mitomycin C sensitivity was measured by counting the surviving colonies. Untreated cells grown in an identical manner served as controls.

### RT-PCR analysis of *ruvA* operon

RNA was isolated from *B. burgdorferi* using RNA-Bee® (Tel-Test, Inc., Friendswood, Texas). *Borrelia* cultures were grown to a concentration of approximately 5×10^7^ organisms/ml. Spirochetes from 1 ml of culture were sedimented by centrifugation and resuspended in 1 ml of RNA-Bee®, and RNA isolation was carried out as per manufacturer's instructions. The resulting RNA was quantitated by UV spectroscopy, checked visually on an agarose gel, and then treated with DNase I (RNase-free, New England Biolabs) following the manufacturer's suggested procedure. Reverse transcription was performed using 200 ng of RNA and primers specific to the *ruvA* operon following the two-step protocol found in the Enhanced Avian HS RT-PCR kit from Sigma-Aldrich. Subsequent PCR reactions used two µl of the resultant cDNA, 25 µM of specific primers and 2X PCR mix from New England Biolabs. Primer sequences and reaction conditions are shown in Table S2.

## Supporting Information

Figure S1 Gene arrangement and transcription patterns of the *ruvAB* locus in the parental clone 5A18NP1 and the *ruvA* mutant T11P01A01. (A) The *ruvAB* locus, with presumed cotranscribed genes *queA* and *pfpB*. The location of the transposon insertion site in the *ruvA* mutant T11P01A01 is shown. (B) RT-PCR analysis of transcription of the *ruvAB* locus of 5A18NP1 and T11P01A01, using the primer pairs shown in (A). RT = with reverse transcriptase, no RT = without reverse transcriptase.
(0.26 MB PDF)

Figure S2 Detailed analysis of the possible recombination events in the *vlsE* variant clonotypes isolated 28 days post inoculation of C3H/HeN mice with the *ruvA* mutant T11P01A01 and the *ruvB* mutant T03TC051. The method of analysis is described in detail in Ref. [11]. Briefly, the horizontal colored bars represent regions of each silent cassette (*vls2* to *vls16*, top to bottom) that may have contributed to sequence changes found in the variant clone. Dark regions in each bar correspond to the regions of sequence changes, whereas the lighter portion of each bar represents the maximal possible region of that silent cassette that could have been exchanged into *vlsE* to produce the observed sequence change. The locations and silent cassette sources of the most likely recombination events are marked by a red dashed box.
(0.65 MB PDF)

Figure S3 Reduced *vlsE* sequence diversity generated following inoculation of C3H/HeN mice with the *ruvB* mutant T03TC051. Clones were isolated from three C3H/HeN mice 28 days post inoculation with 10^5^ T03TC051. The *vlsE* cassette region sequences of each clone were optimally aligned and then analyzed for sequence diversity using a phylogenetic tree program. The groups of clones isolated from each mouse and their tissue source are indicated. Trees are rooted with the 5A18NP1 parental *vlsE* sequence.
(0.08 MB PDF)

Table S1 The *B. burgdorferi ruvA* mutant T11P01A01 and the *ruvB* mutant T03TC051 exhibit decreased *vlsE* sequence variation during infection of C3H/HeN and C3H/SCID mice.
(0.01 MB PDF)

Table S2 Primers used in RT-PCR of the *ruvAB* locus.
(0.01 MB PDF)

Table S3 *B. burgdorferi* isolates generated in this study.
(0.43 MB PDF)
