# Supplementary figures and images for: Central Role of the Holliday Junction Helicase RuvAB in vlsE Recombination and Infectivity of Borrelia burgdorferi
Source: PLoS Pathog. 2009 Dec 4;5(12):e1000679. doi: 10.1371/journal.ppat.1000679 (PMC2780311; doi:10.1371/journal.ppat.1000679)

**A**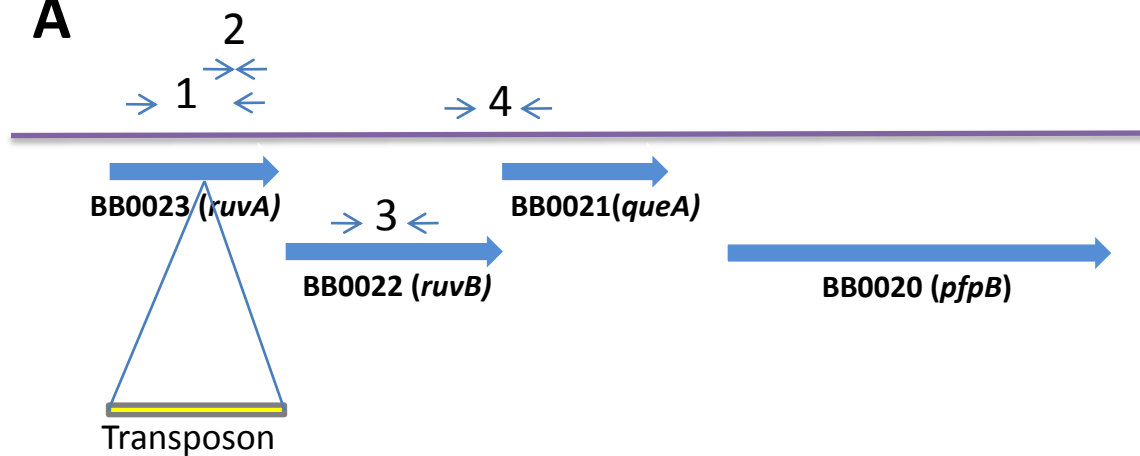**B**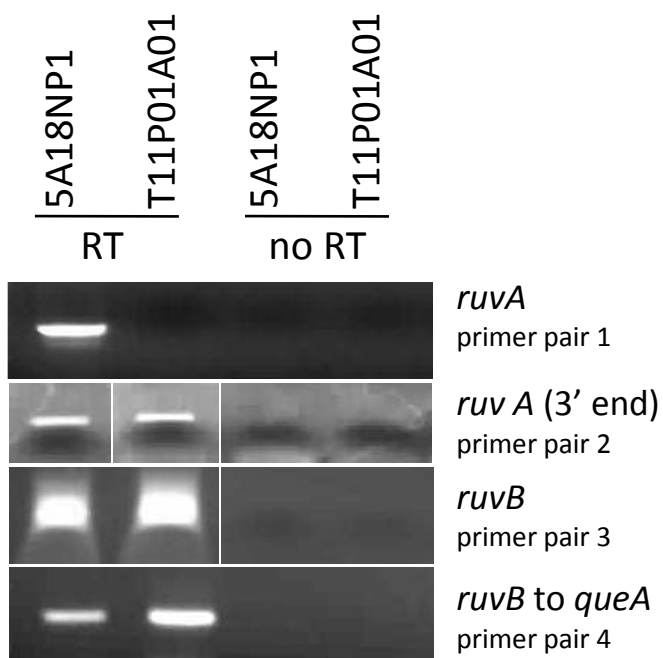

Supplement: Figure S1 — Gene arrangement and transcription patterns of the ruvAB locus in the parental clone 5A18NP1 and the ruvA mutant T11P01A01. (A) The ruvAB locus, with presumed cotranscribed genes queA and pfpB. The location of the transposon insertion site in the ruvA mutant T11P01A01 is shown. (B) RT-PCR analysis of transcription of the ruvAB locus of 5A18NP1 and T11P01A01, using the primer pairs shown in (A). RT = with reverse transcriptase, no RT = without reverse transcriptase. (0.26 MB PDF) [file ppat.1000679.s001.pdf]

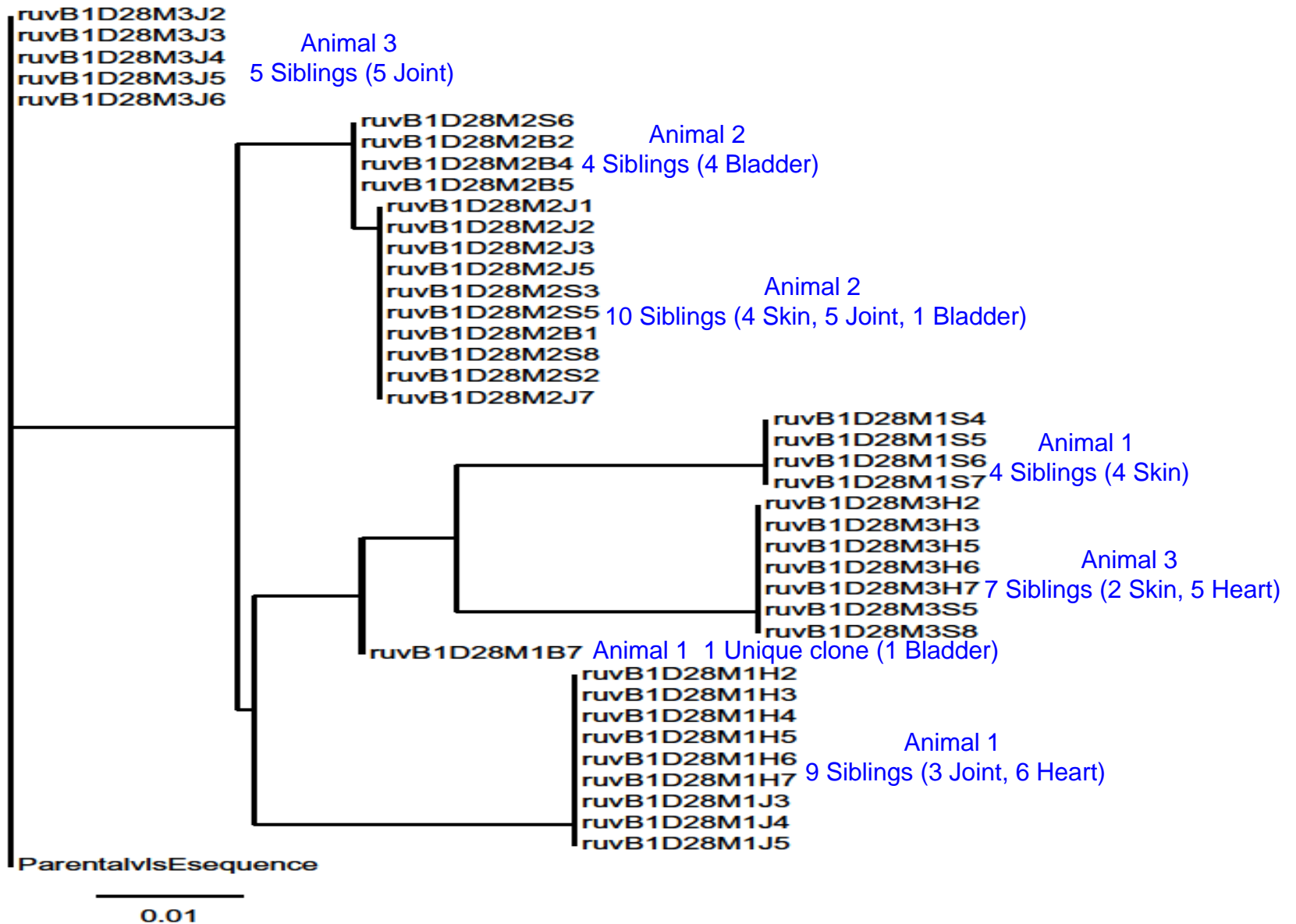

T03TC051. 3 mice shown. One unique sequence, 6 sequences with siblings, and 5 parental sequences

Supplement: Figure S3 — Reduced vlsE sequence diversity generated following inoculation of C3H/HeN mice with the ruvB mutant T03TC051. Clones were isolated from three C3H/HeN mice 28 days post inoculation with 105 T03TC051. The vlsE cassette region sequences of each clone were optimally aligned and then analyzed for sequence diversity using a phylogenetic tree program. The groups of clones isolated from each mouse and their tissue source are indicated. Trees are rooted with the 5A18NP1 parental vlsE sequence. (0.08 MB PDF) [file ppat.1000679.s003.pdf]
