# Supplementary material for: Central Role of the Holliday Junction Helicase RuvAB in vlsE Recombination and Infectivity of Borrelia burgdorferi
Source: PLoS Pathog. 2009 Dec 4;5(12):e1000679. doi: 10.1371/journal.ppat.1000679 (PMC2780311; doi:10.1371/journal.ppat.1000679)
Supplement: Figure S2 — Detailed analysis of the possible recombination events in the vlsE variant clonotypes isolated 28 days post inoculation of C3H/HeN mice with the ruvA mutant T11P01A01 and the ruvB mutant T03TC051. The method of analysis is described in detail in Ref. [11]. Briefly, the horizontal colored bars represent regions of each silent cassette (vls2 to vls16, top to bottom) that may have contributed to sequence changes found in the variant clone. Dark regions in each bar correspond to the regions of sequence changes, whereas the lighter portion of each bar represents the maximal possible region of that silent cassette that could have been exchanged into vlsE to produce the observed sequence change. The locations and silent cassette sources of the most likely recombination events are marked by a red dashed box. (0.65 MB PDF) [file ppat.1000679.s002.pdf]

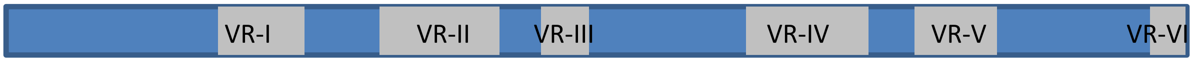

Control-5A18NP1

5A18NP1D28M1B3 – A representative recombinant from infection with 5A18NP1, the parental strain, Silent cassettes 3, 6, 7, 9, 11, and 12 appear to be used.

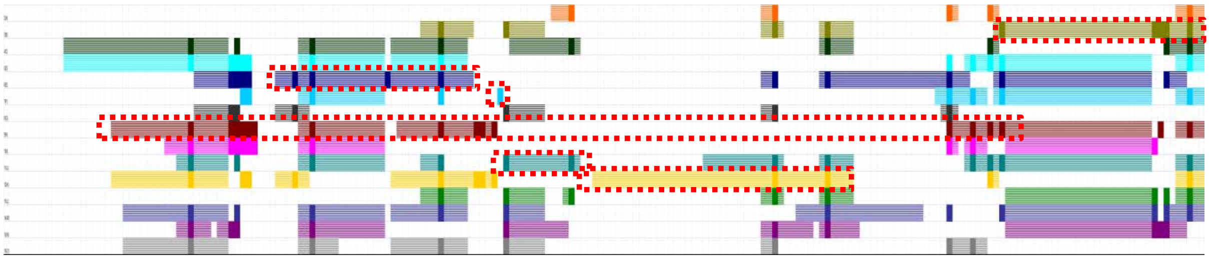

<u>Variants recovered from animals infected with *ruvA* mutant</u>

Animal 1-*ruvA*

ruvA1D28M1S4 – 12 siblings, One large recombination with *vls5* from VR-I to VR-VI

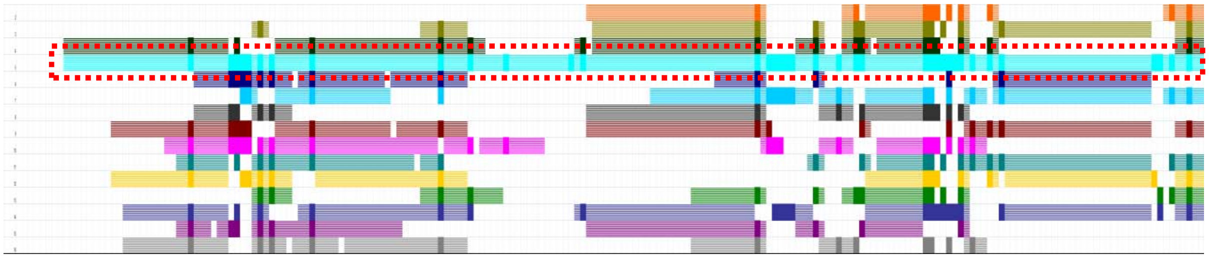

Animal 2-*ruvA*

ruvA1D28M2J1 – 9 siblings, 3 untemplated codon changes but no templated changes

ruvA1D28M2J3 – 8 siblings, same untemplated changes as in ruvA1D28M2J01 +
one large intermittent recombination with *vls2* from VR-III to VR-VI

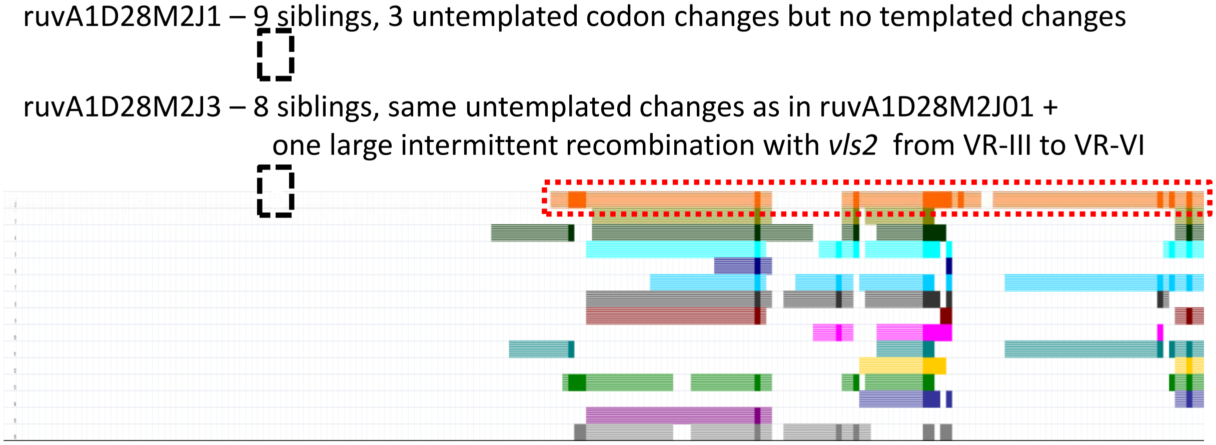

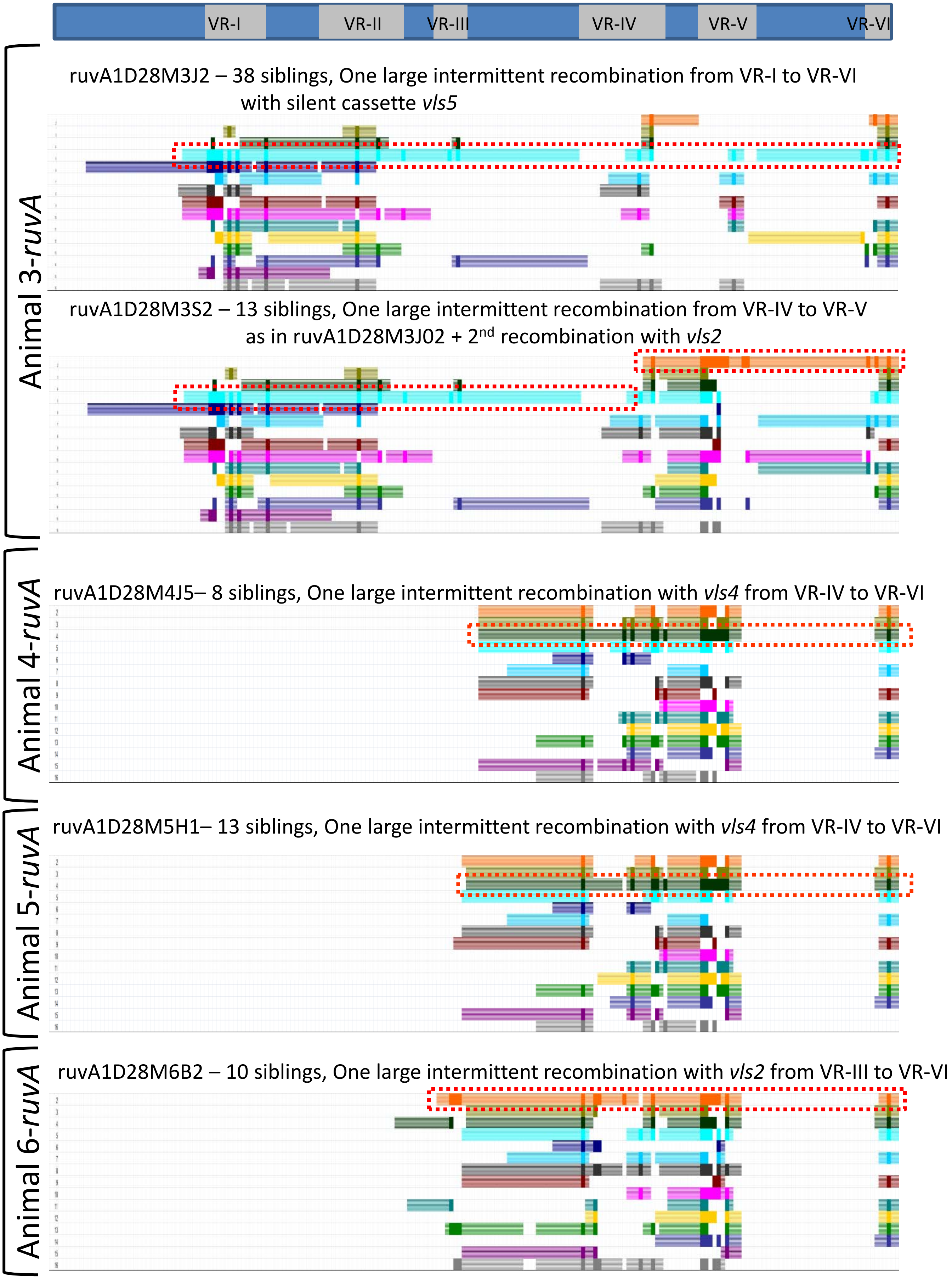

VR-I
VR-II
VR-III
VR-IV
VR-V
VR-VI
Animal 3-*ruvA*
ruvA1D28M3J2 – 38 siblings, One large intermittent recombination from VR-I to VR-VI with silent cassette *vls5*
ruvA1D28M3S2 – 13 siblings, One large intermittent recombination from VR-IV to VR-V as in ruvA1D28M3J02 + 2nd recombination with *vls2*
Animal 4-*ruvA*
ruvA1D28M4J5– 8 siblings, One large intermittent recombination with *vls4* from VR-IV to VR-VI
Animal 5-*ruvA*
ruvA1D28M5H1– 13 siblings, One large intermittent recombination with *vls4* from VR-IV to VR-VI
Animal 6-*ruvA*
ruvA1D28M6B2 – 10 siblings, One large intermittent recombination with *vls2* from VR-III to VR-VI

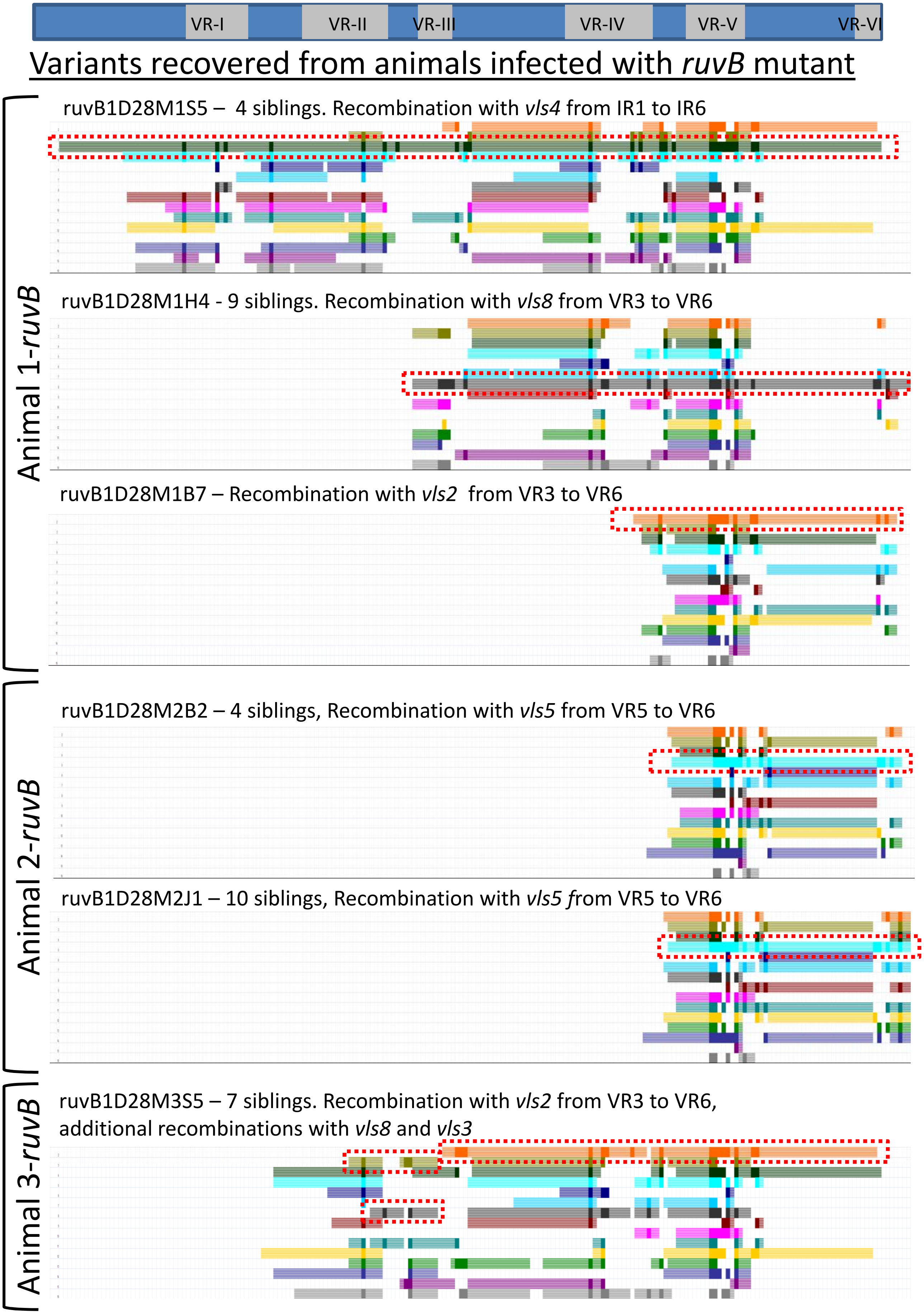

VR-I
VR-II
VR-III
VR-IV
VR-V
VR-VI
Variants recovered from animals infected with *ruvB* mutant
Animal 1-*ruvB*
ruvB1D28M1S5 – 4 siblings. Recombination with *vls4* from IR1 to IR6
ruvB1D28M1H4 - 9 siblings. Recombination with *vls8* from VR3 to VR6
ruvB1D28M1B7 – Recombination with *vls2* from VR3 to VR6
Animal 2-*ruvB*
ruvB1D28M2B2 – 4 siblings, Recombination with *vls5* from VR5 to VR6
ruvB1D28M2J1 – 10 siblings, Recombination with *vls5 f*rom VR5 to VR6
Animal 3-*ruvB*
ruvB1D28M3S5 – 7 siblings. Recombination with *vls2* from VR3 to VR6,
additional recombinations with *vls8* and *vls3*
