# Supplementary material for: Central Role of the Holliday Junction Helicase RuvAB in vlsE Recombination and Infectivity of Borrelia burgdorferi
Source: PLoS Pathog. 2009 Dec 4;5(12):e1000679. doi: 10.1371/journal.ppat.1000679 (PMC2780311; doi:10.1371/journal.ppat.1000679)
Supplement: Table S1 — The B. burgdorferi ruvA mutant T11P01A01 and the ruvB mutant T03TC051 exhibit decreased vlsE sequence variation during infection of C3H/HeN and C3H/SCID mice. (0.01 MB PDF) [file ppat.1000679.s004.pdf]

Table S1. The *B. burgdorferi ruvA* mutant T11P01A01 and the *ruvB* mutant T03TC051 exhibit decreased *vlsE* sequence variation during infection of C3H/HeN and C3H/SCID mice.

B. burgdorferi strain	Day post inoculation	Mouse strain	Total no. of clones sequenced	No. of clones with parental sequence (%)	No. of variants with unique sequence (%)	No. of variants with non-unique sequence (%)	No. of mice tested	No. of tissues tested
*ruv*A mutant	7	C3H/HeN	99	99 (100)	0 (0)	0 (0)	5	5[a]
ruvB mutant	7	C3H/HeN	22	22 (100)	0 (0)	0 (0)	4	4[a]
5A18NP1	7	C3H/HeN	65	7 (11)	38 (58)	20 (31)	5	5[a]
*ruv*A mutant	14	C3H/HeN	16	16 (100)	0 (0)	0 (0)	3	3[b]
5A18NP1	14	C3H/HeN	59	0 (0)	48 (81)	11 (19)	1	3[c]
*ruv*A mutant	28	C3H/HeN	114	3 (3)	0 (0)	111 (97)	6	12[c]
ruvB mutant	28	C3H/HeN	40	5 (12.5)	1 (2.5)	34 (85)	3	10[d]
5A18NP1	28	C3H/HeN	63	0 (0)	31 (49)	32 (51)	1	4[d]
*ruv*A mutant	14	C3H/SCID	85	85 (100)	0 (0)	0 (0)	5	20[d]
5A18NP1	14	C3H/SCID	76	19 (25)	51 (67)	6 (8)	5	17[d]
*ruv*A mutant	28	C3H/SCID	68	68 (100)	0 (0)	0 (0)	5	18[d]
5A18NP1	28	C3H/SCID	47	0 (0)	41 (87)	6 (13)	5	16[d]

[a] skin isolates only
[b] joint isolates only
[c] skin, joint, bladder isolates
[d] skin, joint, heart, and bladder isolates
