# Supplementary material for: Central Role of the Holliday Junction Helicase RuvAB in vlsE Recombination and Infectivity of Borrelia burgdorferi
Source: PLoS Pathog. 2009 Dec 4;5(12):e1000679. doi: 10.1371/journal.ppat.1000679 (PMC2780311; doi:10.1371/journal.ppat.1000679)
Supplement: Table S2 — Primers used in RT-PCR of the ruvAB locus. (0.01 MB PDF) [file ppat.1000679.s005.pdf]

**Table S2. Primers used in RT-PCR of the *ruvAB* locus.**		
**Primer name**	**cDNA target**	**Sequence**
Primer pair 1-forward (5753)	ruvA coding sequence	5’-ACTTTTAGTTAGTGCATTTTGCCTTGC-3’
Primer pair 1-reverse (5756)		5’-TAAAAACTGCTCCTTTTCAGAATC-3’
Primer pair 2-forward (5755)	ruvA coding sequence 3’ of transposon insertion site	5’-GCGAGCTTGAATCCACTGGTC-3’
Primer pair 2-reverse (5756)		5’-TAAAAACTGCTCCTTTTCAGAATC-3’
Primer pair 3-forward (5757)	ruvB coding sequence	5’-GAGAGAGATGAGGCTCTAGATCATG-3’
Primer pair 3-reverse (5758)		5’-GAAATAGCTAAAGTATCAACACCTAC-3’
Primer pair 4-forward (5781)	ruvB to queA coding sequence	5’-GTAGGTGTTGATACTTTAGCTATTTC-3’
Primer pair 4-reverse (5782)		5’-GCACGATTAACCTTGAAGATCCTC-3’
