# Supplementary material for: Central Role of the Holliday Junction Helicase RuvAB in vlsE Recombination and Infectivity of Borrelia burgdorferi
Source: PLoS Pathog. 2009 Dec 4;5(12):e1000679. doi: 10.1371/journal.ppat.1000679 (PMC2780311; doi:10.1371/journal.ppat.1000679)
Supplement: Table S3 — B. burgdorferi isolates generated in this study. (0.43 MB PDF) [file ppat.1000679.s006.pdf]

Table S3. *B. burgdorferi* isolates generated in this study.
Parental = identical to *vlsE* cassette region sequence of 5A18NP1 (parental strain)

Clone Name	GenBank No.	Infecting Clone	Mouse Strain	Days Post Infection	Tissue
vlsE cassette region sequence of 5A18NP1	GQ369288	--	--	--	--
5A18NP1D7M1S02	GQ506180	5A18NP1	C3H/HeN	D7	Skin
5A18NP1D7M1S06	GQ506181	5A18NP1	C3H/HeN	D7	Skin
5A18NP1D7M1S08	GQ506182	5A18NP1	C3H/HeN	D7	Skin
5A18NP1D7M1S11	GQ506183	5A18NP1	C3H/HeN	D7	Skin
5A18NP1D7M1S12	GQ506184	5A18NP1	C3H/HeN	D7	Skin
5A18NP1D7M1S13	GQ506185	5A18NP1	C3H/HeN	D7	Skin
5A18NP1D7M1S14	GQ506186	5A18NP1	C3H/HeN	D7	Skin
5A18NP1D7M1S15	GQ506187	5A18NP1	C3H/HeN	D7	Skin
5A18NP1D7M1S16	GQ506188	5A18NP1	C3H/HeN	D7	Skin
5A18NP1D7M1S18	GQ506189	5A18NP1	C3H/HeN	D7	Skin
5A18NP1D7M1S19	GQ506190	5A18NP1	C3H/HeN	D7	Skin
5A18NP1D7M1S20	GQ506191	5A18NP1	C3H/HeN	D7	Skin
5A18NP1D7M1S23	GQ506192	5A18NP1	C3H/HeN	D7	Skin
5A18NP1D7M1S24	GQ506193	5A18NP1	C3H/HeN	D7	Skin
5A18NP1D7M2S06	GQ506155	5A18NP1	C3H/HeN	D7	Skin
5A18NP1D7M2S11	GQ506156	5A18NP1	C3H/HeN	D7	Skin
5A18NP1D7M2S12	GQ506157	5A18NP1	C3H/HeN	D7	Skin
5A18NP1D7M2S14	GQ506158	5A18NP1	C3H/HeN	D7	Skin
5A18NP1D7M2S17	Parental	5A18NP1	C3H/HeN	D7	Skin
5A18NP1D7M2S18	GQ506160	5A18NP1	C3H/HeN	D7	Skin
5A18NP1D7M2S20	GQ506161	5A18NP1	C3H/HeN	D7	Skin
5A18NP1D7M2S23	GQ506162	5A18NP1	C3H/HeN	D7	Skin
5A18NP1D7M2S24	GQ506163	5A18NP1	C3H/HeN	D7	Skin
5A18NP1D7M3S01	GQ506141	5A18NP1	C3H/HeN	D7	Skin
5A18NP1D7M3S02	GQ506142	5A18NP1	C3H/HeN	D7	Skin
5A18NP1D7M3S03	GQ506143	5A18NP1	C3H/HeN	D7	Skin
5A18NP1D7M3S04	GQ506144	5A18NP1	C3H/HeN	D7	Skin
5A18NP1D7M3S05	GQ506145	5A18NP1	C3H/HeN	D7	Skin
5A18NP1D7M3S06	Parental	5A18NP1	C3H/HeN	D7	Skin
5A18NP1D7M3S07	GQ506147	5A18NP1	C3H/HeN	D7	Skin
5A18NP1D7M3S11	GQ506148	5A18NP1	C3H/HeN	D7	Skin
5A18NP1D7M3S14	GQ506149	5A18NP1	C3H/HeN	D7	Skin
5A18NP1D7M3S15	Parental	5A18NP1	C3H/HeN	D7	Skin
5A18NP1D7M3S17	GQ506151	5A18NP1	C3H/HeN	D7	Skin
5A18NP1D7M3S18	GQ506152	5A18NP1	C3H/HeN	D7	Skin
5A18NP1D7M3S20	Parental	5A18NP1	C3H/HeN	D7	Skin
5A18NP1D7M3S22	GQ506154	5A18NP1	C3H/HeN	D7	Skin
5A18NP1D7M4S01	GQ506164	5A18NP1	C3H/HeN	D7	Skin
5A18NP1D7M4S03	GQ506165	5A18NP1	C3H/HeN	D7	Skin
5A18NP1D7M4S04	GQ506166	5A18NP1	C3H/HeN	D7	Skin

5A18NP1D7M4S05	GQ506167	5A18NP1	C3H/HeN	D7	Skin
5A18NP1D7M4S08	GQ506168	5A18NP1	C3H/HeN	D7	Skin
5A18NP1D7M4S09	GQ506169	5A18NP1	C3H/HeN	D7	Skin
5A18NP1D7M4S10	GQ506170	5A18NP1	C3H/HeN	D7	Skin
5A18NP1D7M4S11	Parental	5A18NP1	C3H/HeN	D7	Skin
5A18NP1D7M4S12	Parental	5A18NP1	C3H/HeN	D7	Skin
5A18NP1D7M4S13	GQ506173	5A18NP1	C3H/HeN	D7	Skin
5A18NP1D7M4S16	GQ506174	5A18NP1	C3H/HeN	D7	Skin
5A18NP1D7M4S18	GQ506175	5A18NP1	C3H/HeN	D7	Skin
5A18NP1D7M4S20	GQ506176	5A18NP1	C3H/HeN	D7	Skin
5A18NP1D7M4S21	GQ506177	5A18NP1	C3H/HeN	D7	Skin
5A18NP1D7M4S23	GQ506178	5A18NP1	C3H/HeN	D7	Skin
5A18NP1D7M4S24	GQ506179	5A18NP1	C3H/HeN	D7	Skin
5A18NP1D7M5S01	GQ506194	5A18NP1	C3H/HeN	D7	Skin
5A18NP1D7M5S02	GQ506195	5A18NP1	C3H/HeN	D7	Skin
5A18NP1D7M5S03	GQ506196	5A18NP1	C3H/HeN	D7	Skin
5A18NP1D7M5S04	GQ506197	5A18NP1	C3H/HeN	D7	Skin
5A18NP1D7M5S05	GQ506198	5A18NP1	C3H/HeN	D7	Skin
5A18NP1D7M5S06	GQ506199	5A18NP1	C3H/HeN	D7	Skin
5A18NP1D7M5S08	Parental	5A18NP1	C3H/HeN	D7	Skin
5A18NP1D7M5S09	GQ506201	5A18NP1	C3H/HeN	D7	Skin
5A18NP1D7M5S10	GQ506202	5A18NP1	C3H/HeN	D7	Skin
5A18NP1D7M5S11	GQ506203	5A18NP1	C3H/HeN	D7	Skin
5A18NP1D7M5S12	GQ506204	5A18NP1	C3H/HeN	D7	Skin
5A18NP1D7M5S13	GQ506205	5A18NP1	C3H/HeN	D7	Skin
5A18NP1D14M1B01	GQ506215	5A18NP1	C3H/HeN	D14	Bladder
5A18NP1D14M1B03	GQ506221	5A18NP1	C3H/HeN	D14	Bladder
5A18NP1D14M1B04	GQ506222	5A18NP1	C3H/HeN	D14	Bladder
5A18NP1D14M1B05	GQ506223	5A18NP1	C3H/HeN	D14	Bladder
5A18NP1D14M1B06	GQ506224	5A18NP1	C3H/HeN	D14	Bladder
5A18NP1D14M1B07	GQ506225	5A18NP1	C3H/HeN	D14	Bladder
5A18NP1D14M1B08	GQ506226	5A18NP1	C3H/HeN	D14	Bladder
5A18NP1D14M1B09	GQ506227	5A18NP1	C3H/HeN	D14	Bladder
5A18NP1D14M1B10	GQ506206	5A18NP1	C3H/HeN	D14	Bladder
5A18NP1D14M1B11	GQ506207	5A18NP1	C3H/HeN	D14	Bladder
5A18NP1D14M1B12	GQ506208	5A18NP1	C3H/HeN	D14	Bladder
5A18NP1D14M1B13	GQ506209	5A18NP1	C3H/HeN	D14	Bladder
5A18NP1D14M1B15	GQ506210	5A18NP1	C3H/HeN	D14	Bladder
5A18NP1D14M1B16	GQ506211	5A18NP1	C3H/HeN	D14	Bladder
5A18NP1D14M1B17	GQ506212	5A18NP1	C3H/HeN	D14	Bladder
5A18NP1D14M1B18	GQ506213	5A18NP1	C3H/HeN	D14	Bladder
5A18NP1D14M1B19	GQ506214	5A18NP1	C3H/HeN	D14	Bladder
5A18NP1D14M1B20	GQ506216	5A18NP1	C3H/HeN	D14	Bladder
5A18NP1D14M1B21	GQ506217	5A18NP1	C3H/HeN	D14	Bladder
5A18NP1D14M1B22	GQ506218	5A18NP1	C3H/HeN	D14	Bladder
5A18NP1D14M1B23	GQ506219	5A18NP1	C3H/HeN	D14	Bladder
5A18NP1D14M1B24	GQ506220	5A18NP1	C3H/HeN	D14	Bladder

5A18NP1D14M1J02	GQ506240	5A18NP1	C3H/HeN	D14	Joint
5A18NP1D14M1J03	GQ506241	5A18NP1	C3H/HeN	D14	Joint
5A18NP1D14M1J04	GQ506242	5A18NP1	C3H/HeN	D14	Joint
5A18NP1D14M1J06	GQ506243	5A18NP1	C3H/HeN	D14	Joint
5A18NP1D14M1J07	GQ506244	5A18NP1	C3H/HeN	D14	Joint
5A18NP1D14M1J08	GQ506245	5A18NP1	C3H/HeN	D14	Joint
5A18NP1D14M1J09	GQ506246	5A18NP1	C3H/HeN	D14	Joint
5A18NP1D14M1J11	GQ506228	5A18NP1	C3H/HeN	D14	Joint
5A18NP1D14M1J12	GQ506229	5A18NP1	C3H/HeN	D14	Joint
5A18NP1D14M1J13	GQ506230	5A18NP1	C3H/HeN	D14	Joint
5A18NP1D14M1J14	GQ506231	5A18NP1	C3H/HeN	D14	Joint
5A18NP1D14M1J15	GQ506232	5A18NP1	C3H/HeN	D14	Joint
5A18NP1D14M1J16	GQ506233	5A18NP1	C3H/HeN	D14	Joint
5A18NP1D14M1J17	GQ506234	5A18NP1	C3H/HeN	D14	Joint
5A18NP1D14M1J19	GQ506235	5A18NP1	C3H/HeN	D14	Joint
5A18NP1D14M1J20	GQ506236	5A18NP1	C3H/HeN	D14	Joint
5A18NP1D14M1J22	GQ506237	5A18NP1	C3H/HeN	D14	Joint
5A18NP1D14M1J23	GQ506238	5A18NP1	C3H/HeN	D14	Joint
5A18NP1D14M1J24	GQ506239	5A18NP1	C3H/HeN	D14	Joint
5A18NP1D14M1S01	GQ506254	5A18NP1	C3H/HeN	D14	Skin
5A18NP1D14M1S02	GQ506259	5A18NP1	C3H/HeN	D14	Skin
5A18NP1D14M1S03	GQ506260	5A18NP1	C3H/HeN	D14	Skin
5A18NP1D14M1S04	GQ506261	5A18NP1	C3H/HeN	D14	Skin
5A18NP1D14M1S05	GQ506262	5A18NP1	C3H/HeN	D14	Skin
5A18NP1D14M1S08	GQ506263	5A18NP1	C3H/HeN	D14	Skin
5A18NP1D14M1S09	GQ506264	5A18NP1	C3H/HeN	D14	Skin
5A18NP1D14M1S10	GQ506247	5A18NP1	C3H/HeN	D14	Skin
5A18NP1D14M1S12	GQ506248	5A18NP1	C3H/HeN	D14	Skin
5A18NP1D14M1S14	GQ506249	5A18NP1	C3H/HeN	D14	Skin
5A18NP1D14M1S15	GQ506250	5A18NP1	C3H/HeN	D14	Skin
5A18NP1D14M1S16	GQ506251	5A18NP1	C3H/HeN	D14	Skin
5A18NP1D14M1S17	GQ506252	5A18NP1	C3H/HeN	D14	Skin
5A18NP1D14M1S18	GQ506253	5A18NP1	C3H/HeN	D14	Skin
5A18NP1D14M1S20	GQ506255	5A18NP1	C3H/HeN	D14	Skin
5A18NP1D14M1S21	GQ506256	5A18NP1	C3H/HeN	D14	Skin
5A18NP1D14M1S22	GQ506257	5A18NP1	C3H/HeN	D14	Skin
5A18NP1D14M1S24	GQ506258	5A18NP1	C3H/HeN	D14	Skin
5A18NP1D28M1B01	GQ506265	5A18NP1	C3H/HeN	D28	Bladder
5A18NP1D28M1B02	GQ506266	5A18NP1	C3H/HeN	D28	Bladder
5A18NP1D28M1B03	GQ506267	5A18NP1	C3H/HeN	D28	Bladder
5A18NP1D28M1B04	GQ506268	5A18NP1	C3H/HeN	D28	Bladder
5A18NP1D28M1B06	GQ506269	5A18NP1	C3H/HeN	D28	Bladder
5A18NP1D28M1B08	GQ506270	5A18NP1	C3H/HeN	D28	Bladder
5A18NP1D28M1B10	GQ506271	5A18NP1	C3H/HeN	D28	Bladder
5A18NP1D28M1B12	GQ506272	5A18NP1	C3H/HeN	D28	Bladder
5A18NP1D28M1B13	GQ506273	5A18NP1	C3H/HeN	D28	Bladder
5A18NP1D28M1B14	GQ506274	5A18NP1	C3H/HeN	D28	Bladder

5A18NP1D28M1B15	GQ506275	5A18NP1	C3H/HeN	D28	Bladder
5A18NP1D28M1B16	GQ506276	5A18NP1	C3H/HeN	D28	Bladder
5A18NP1D28M1B18	GQ506277	5A18NP1	C3H/HeN	D28	Bladder
5A18NP1D28M1B19	GQ506278	5A18NP1	C3H/HeN	D28	Bladder
5A18NP1D28M1B20	GQ506279	5A18NP1	C3H/HeN	D28	Bladder
5A18NP1D28M1B21	GQ506280	5A18NP1	C3H/HeN	D28	Bladder
5A18NP1D28M1B22	GQ506281	5A18NP1	C3H/HeN	D28	Bladder
5A18NP1D28M1B24	GQ506282	5A18NP1	C3H/HeN	D28	Bladder
5A18NP1D28M1H02	GQ506283	5A18NP1	C3H/HeN	D28	Heart
5A18NP1D28M1H03	GQ506284	5A18NP1	C3H/HeN	D28	Heart
5A18NP1D28M1H05	GQ506285	5A18NP1	C3H/HeN	D28	Heart
5A18NP1D28M1H06	GQ506286	5A18NP1	C3H/HeN	D28	Heart
5A18NP1D28M1H07	GQ506287	5A18NP1	C3H/HeN	D28	Heart
5A18NP1D28M1H08	GQ506288	5A18NP1	C3H/HeN	D28	Heart
5A18NP1D28M1H09	GQ506289	5A18NP1	C3H/HeN	D28	Heart
5A18NP1D28M1H10	GQ506290	5A18NP1	C3H/HeN	D28	Heart
5A18NP1D28M1H11	GU067769	5A18NP1	C3H/HeN	D28	Heart
5A18NP1D28M1H12	GQ506291	5A18NP1	C3H/HeN	D28	Heart
5A18NP1D28M1H13	GQ506292	5A18NP1	C3H/HeN	D28	Heart
5A18NP1D28M1H14	GQ506293	5A18NP1	C3H/HeN	D28	Heart
5A18NP1D28M1H15	GQ506294	5A18NP1	C3H/HeN	D28	Heart
5A18NP1D28M1H16	GQ506295	5A18NP1	C3H/HeN	D28	Heart
5A18NP1D28M1H18	GQ506296	5A18NP1	C3H/HeN	D28	Heart
5A18NP1D28M1H19	GQ506297	5A18NP1	C3H/HeN	D28	Heart
5A18NP1D28M1H20	GQ506298	5A18NP1	C3H/HeN	D28	Heart
5A18NP1D28M1H21	GQ506299	5A18NP1	C3H/HeN	D28	Heart
5A18NP1D28M1H22	GQ506300	5A18NP1	C3H/HeN	D28	Heart
5A18NP1D28M1H24	GQ506301	5A18NP1	C3H/HeN	D28	Heart
5A18NP1D28M1J04	GQ506302	5A18NP1	C3H/HeN	D28	Joint
5A18NP1D28M1J05	GQ506303	5A18NP1	C3H/HeN	D28	Joint
5A18NP1D28M1J06	GQ506304	5A18NP1	C3H/HeN	D28	Joint
5A18NP1D28M1J07	GQ506305	5A18NP1	C3H/HeN	D28	Joint
5A18NP1D28M1J09	GQ506306	5A18NP1	C3H/HeN	D28	Joint
5A18NP1D28M1J10	GQ506307	5A18NP1	C3H/HeN	D28	Joint
5A18NP1D28M1J13	GQ506308	5A18NP1	C3H/HeN	D28	Joint
5A18NP1D28M1J14	GQ506309	5A18NP1	C3H/HeN	D28	Joint
5A18NP1D28M1J15	GQ506310	5A18NP1	C3H/HeN	D28	Joint
5A18NP1D28M1J16	GQ506311	5A18NP1	C3H/HeN	D28	Joint
5A18NP1D28M1J19	GQ506312	5A18NP1	C3H/HeN	D28	Joint
5A18NP1D28M1J20	GQ506313	5A18NP1	C3H/HeN	D28	Joint
5A18NP1D28M1J21	GQ506314	5A18NP1	C3H/HeN	D28	Joint
5A18NP1D28M1J22	GQ506315	5A18NP1	C3H/HeN	D28	Joint
5A18NP1D28M1J25	GQ506316	5A18NP1	C3H/HeN	D28	Joint
5A18NP1D28M1S02	GQ506317	5A18NP1	C3H/HeN	D28	Skin
5A18NP1D28M1S05	GU067770	5A18NP1	C3H/HeN	D28	Skin
5A18NP1D28M1S08	GQ506318	5A18NP1	C3H/HeN	D28	Skin
5A18NP1D28M1S10	GQ506319	5A18NP1	C3H/HeN	D28	Skin

5A18NP1D28M1S15	GQ506320	5A18NP1	C3H/HeN	D28	Skin
5A18NP1D28M1S16	GQ506321	5A18NP1	C3H/HeN	D28	Skin
5A18NP1D28M1S18	GQ506322	5A18NP1	C3H/HeN	D28	Skin
5A18NP1D28M1S19	GQ506323	5A18NP1	C3H/HeN	D28	Skin
5A18NP1D28M1S21	GQ506324	5A18NP1	C3H/HeN	D28	Skin
5A18NP1D28M1S23	GQ506325	5A18NP1	C3H/HeN	D28	Skin
5A18NP1SD14M1B1	Parental	5A18NP1	C3H/scid	D14	Bladder
5A18NP1SD14M1B2	Parental	5A18NP1	C3H/scid	D14	Bladder
5A18NP1SD14M1B3	GQ506346	5A18NP1	C3H/scid	D14	Bladder
5A18NP1SD14M1B4	Parental	5A18NP1	C3H/scid	D14	Bladder
5A18NP1SD14M1B5	Parental	5A18NP1	C3H/scid	D14	Bladder
5A18NP1SD14M1B6	GQ506349	5A18NP1	C3H/scid	D14	Bladder
5A18NP1SD14M1J1	Parental	5A18NP1	C3H/scid	D14	Joint
5A18NP1SD14M1J3	GQ506351	5A18NP1	C3H/scid	D14	Joint
5A18NP1SD14M1S2	Parental	5A18NP1	C3H/scid	D14	Skin
5A18NP1SD14M1S3	GQ506353	5A18NP1	C3H/scid	D14	Skin
5A18NP1SD14M1S4	Parental	5A18NP1	C3H/scid	D14	Skin
5A18NP1SD14M1S5	GQ506355	5A18NP1	C3H/scid	D14	Skin
5A18NP1SD14M1S6	GQ506356	5A18NP1	C3H/scid	D14	Skin
5A18NP1SD14M2B1	GQ506369	5A18NP1	C3H/scid	D14	Bladder
5A18NP1SD14M2B3	GQ506370	5A18NP1	C3H/scid	D14	Bladder
5A18NP1SD14M2B4	GQ506371	5A18NP1	C3H/scid	D14	Bladder
5A18NP1SD14M2B5	Parental	5A18NP1	C3H/scid	D14	Bladder
5A18NP1SD14M2B6	GQ506373	5A18NP1	C3H/scid	D14	Bladder
5A18NP1SD14M2H1	GQ506374	5A18NP1	C3H/scid	D14	Heart
5A18NP1SD14M2H2	GQ506375	5A18NP1	C3H/scid	D14	Heart
5A18NP1SD14M2H3	GQ506376	5A18NP1	C3H/scid	D14	Heart
5A18NP1SD14M2H4	GQ506377	5A18NP1	C3H/scid	D14	Heart
5A18NP1SD14M2H5	GQ506378	5A18NP1	C3H/scid	D14	Heart
5A18NP1SD14M2J1	GQ506379	5A18NP1	C3H/scid	D14	Joint
5A18NP1SD14M2J2	GQ506380	5A18NP1	C3H/scid	D14	Joint
5A18NP1SD14M2J3	GQ506381	5A18NP1	C3H/scid	D14	Joint
5A18NP1SD14M2J4	Parental	5A18NP1	C3H/scid	D14	Joint
5A18NP1SD14M2J5	GQ506383	5A18NP1	C3H/scid	D14	Joint
5A18NP1SD14M2J6	Parental	5A18NP1	C3H/scid	D14	Joint
5A18NP1SD14M2S1	GQ506385	5A18NP1	C3H/scid	D14	Skin
5A18NP1SD14M2S2	Parental	5A18NP1	C3H/scid	D14	Skin
5A18NP1SD14M2S3	GQ506387	5A18NP1	C3H/scid	D14	Skin
5A18NP1SD14M2S4	Parental	5A18NP1	C3H/scid	D14	Skin
5A18NP1SD14M2S5	GQ506389	5A18NP1	C3H/scid	D14	Skin
5A18NP1SD14M2S6	Parental	5A18NP1	C3H/scid	D14	Skin
5A18NP1SD14M3B1	GQ506326	5A18NP1	C3H/scid	D14	Bladder
5A18NP1SD14M3B3	GQ506327	5A18NP1	C3H/scid	D14	Bladder
5A18NP1SD14M3B4	GQ506328	5A18NP1	C3H/scid	D14	Bladder
5A18NP1SD14M3B5	GQ506329	5A18NP1	C3H/scid	D14	Bladder
5A18NP1SD14M3B6	GQ506330	5A18NP1	C3H/scid	D14	Bladder
5A18NP1SD14M3H1	GQ506331	5A18NP1	C3H/scid	D14	Heart

5A18NP1SD14M3H2	GQ506332	5A18NP1	C3H/scid	D14	Heart
5A18NP1SD14M3H3	GQ506333	5A18NP1	C3H/scid	D14	Heart
5A18NP1SD14M3H4	GQ506334	5A18NP1	C3H/scid	D14	Heart
5A18NP1SD14M3H5	GQ506335	5A18NP1	C3H/scid	D14	Heart
5A18NP1SD14M3H6	GQ506336	5A18NP1	C3H/scid	D14	Heart
5A18NP1SD14M3J1	GQ506337	5A18NP1	C3H/scid	D14	Joint
5A18NP1SD14M3J2	Parental	5A18NP1	C3H/scid	D14	Joint
5A18NP1SD14M3J3	GQ506339	5A18NP1	C3H/scid	D14	Joint
5A18NP1SD14M3J5	GQ506340	5A18NP1	C3H/scid	D14	Joint
5A18NP1SD14M3J6	GQ506341	5A18NP1	C3H/scid	D14	Joint
5A18NP1SD14M3S2	GQ506342	5A18NP1	C3H/scid	D14	Skin
5A18NP1SD14M3S5	Parental	5A18NP1	C3H/scid	D14	Skin
5A18NP1SD14M4B1	GQ506357	5A18NP1	C3H/scid	D14	Bladder
5A18NP1SD14M4B4	GQ506358	5A18NP1	C3H/scid	D14	Bladder
5A18NP1SD14M4B5	GQ506359	5A18NP1	C3H/scid	D14	Bladder
5A18NP1SD14M4B6	GQ506360	5A18NP1	C3H/scid	D14	Bladder
5A18NP1SD14M4J1	GQ506361	5A18NP1	C3H/scid	D14	Joint
5A18NP1SD14M4J2	Parental	5A18NP1	C3H/scid	D14	Joint
5A18NP1SD14M4J3	GQ506363	5A18NP1	C3H/scid	D14	Joint
5A18NP1SD14M4J5	GQ506364	5A18NP1	C3H/scid	D14	Joint
5A18NP1SD14M4S1	GQ506365	5A18NP1	C3H/scid	D14	Skin
5A18NP1SD14M4S2	GQ506366	5A18NP1	C3H/scid	D14	Skin
5A18NP1SD14M4S3	GQ506367	5A18NP1	C3H/scid	D14	Skin
5A18NP1SD14M4S6	Parental	5A18NP1	C3H/scid	D14	Skin
5A18NP1SD14M5H1	GQ506398	5A18NP1	C3H/scid	D14	Heart
5A18NP1SD14M5H2	GQ506399	5A18NP1	C3H/scid	D14	Heart
5A18NP1SD14M5H5	GQ506400	5A18NP1	C3H/scid	D14	Heart
5A18NP1SD14M5H6	GQ506401	5A18NP1	C3H/scid	D14	Heart
5A18NP1SD14M5J1	Parental	5A18NP1	C3H/scid	D14	Joint
5A18NP1SD14M5J3	GQ506392	5A18NP1	C3H/scid	D14	Joint
5A18NP1SD14M5J4	GQ506393	5A18NP1	C3H/scid	D14	Joint
5A18NP1SD14M5S2	Parental	5A18NP1	C3H/scid	D14	Skin
5A18NP1SD14M5S3	GQ506395	5A18NP1	C3H/scid	D14	Skin
5A18NP1SD14M5S4	GQ506396	5A18NP1	C3H/scid	D14	Skin
5A18NP1SD14M5S5	GQ506397	5A18NP1	C3H/scid	D14	Skin
5A18NP1SD28M1B1	GQ506407	5A18NP1	C3H/scid	D28	Bladder
5A18NP1SD28M1B4	GQ506408	5A18NP1	C3H/scid	D28	Bladder
5A18NP1SD28M1B6	GQ506409	5A18NP1	C3H/scid	D28	Bladder
5A18NP1SD28M1H1	GQ506410	5A18NP1	C3H/scid	D28	Heart
5A18NP1SD28M1H2	GQ506411	5A18NP1	C3H/scid	D28	Heart
5A18NP1SD28M1H4	GQ506412	5A18NP1	C3H/scid	D28	Heart
5A18NP1SD28M1J1	GQ506413	5A18NP1	C3H/scid	D28	Joint
5A18NP1SD28M1J2	GQ506414	5A18NP1	C3H/scid	D28	Joint
5A18NP1SD28M1J6	GQ506415	5A18NP1	C3H/scid	D28	Joint
5A18NP1SD28M1S3	GQ506416	5A18NP1	C3H/scid	D28	Skin
5A18NP1SD28M1S6	GQ506417	5A18NP1	C3H/scid	D28	Skin
5A18NP1SD28M2B2	GQ506427	5A18NP1	C3H/scid	D28	Bladder

5A18NP1SD28M2H3	GQ506428	5A18NP1	C3H/scid	D28	Heart
5A18NP1SD28M2J1	GQ506429	5A18NP1	C3H/scid	D28	Joint
5A18NP1SD28M2J2	GQ506430	5A18NP1	C3H/scid	D28	Joint
5A18NP1SD28M2J3	GQ506431	5A18NP1	C3H/scid	D28	Joint
5A18NP1SD28M2J4	GQ506432	5A18NP1	C3H/scid	D28	Joint
5A18NP1SD28M2J5	GQ506433	5A18NP1	C3H/scid	D28	Joint
5A18NP1SD28M2J6	GQ506434	5A18NP1	C3H/scid	D28	Joint
5A18NP1SD28M2S1	GQ506435	5A18NP1	C3H/scid	D28	Skin
5A18NP1SD28M2S2	GQ506436	5A18NP1	C3H/scid	D28	Skin
5A18NP1SD28M2S3	GQ506437	5A18NP1	C3H/scid	D28	Skin
5A18NP1SD28M2S4	GQ506438	5A18NP1	C3H/scid	D28	Skin
5A18NP1SD28M2S5	GQ506439	5A18NP1	C3H/scid	D28	Skin
5A18NP1SD28M2S6	GQ506440	5A18NP1	C3H/scid	D28	Skin
5A18NP1SD28M3J2	GQ506406	5A18NP1	C3H/scid	D28	Joint
5A18NP1SD28M3S2	GQ506402	5A18NP1	C3H/scid	D28	Skin
5A18NP1SD28M3S3	GQ506403	5A18NP1	C3H/scid	D28	Skin
5A18NP1SD28M3S4	GQ506404	5A18NP1	C3H/scid	D28	Skin
5A18NP1SD28M3S6	GQ506405	5A18NP1	C3H/scid	D28	Skin
5A18NP1SD28M4B1	GQ506418	5A18NP1	C3H/scid	D28	Bladder
5A18NP1SD28M4B3	GQ506419	5A18NP1	C3H/scid	D28	Bladder
5A18NP1SD28M4B4	GQ506420	5A18NP1	C3H/scid	D28	Bladder
5A18NP1SD28M4J1	GQ506421	5A18NP1	C3H/scid	D28	Joint
5A18NP1SD28M4J4	GQ506422	5A18NP1	C3H/scid	D28	Joint
5A18NP1SD28M4J6	GQ506423	5A18NP1	C3H/scid	D28	Joint
5A18NP1SD28M4S2	GQ506424	5A18NP1	C3H/scid	D28	Skin
5A18NP1SD28M4S4	GQ506425	5A18NP1	C3H/scid	D28	Skin
5A18NP1SD28M4S5	GQ506426	5A18NP1	C3H/scid	D28	Skin
5A18NP1SD28M5B1	GQ506441	5A18NP1	C3H/scid	D28	Bladder
5A18NP1SD28M5B3	GQ506442	5A18NP1	C3H/scid	D28	Bladder
5A18NP1SD28M5B8	GQ506443	5A18NP1	C3H/scid	D28	Bladder
5A18NP1SD28M5J1	GQ506444	5A18NP1	C3H/scid	D28	Joint
5A18NP1SD28M5J5	GQ506445	5A18NP1	C3H/scid	D28	Joint
5A18NP1SD28M5S1	GQ506446	5A18NP1	C3H/scid	D28	Skin
5A18NP1SD28M5S3	GQ506447	5A18NP1	C3H/scid	D28	Skin
5A18NP1SD28M5S4	GQ506448	5A18NP1	C3H/scid	D28	Skin
ruvA1D7M1S02	Parental	T11P01A01	C3H/HeN	D7	Skin
ruvA1D7M1S04	Parental	T11P01A01	C3H/HeN	D7	Skin
ruvA1D7M1S05	Parental	T11P01A01	C3H/HeN	D7	Skin
ruvA1D7M1S07	Parental	T11P01A01	C3H/HeN	D7	Skin
ruvA1D7M1S09	Parental	T11P01A01	C3H/HeN	D7	Skin
ruvA1D7M1S11	Parental	T11P01A01	C3H/HeN	D7	Skin
ruvA1D7M1S12	Parental	T11P01A01	C3H/HeN	D7	Skin
ruvA1D7M1S14	Parental	T11P01A01	C3H/HeN	D7	Skin
ruvA1D7M1S15	Parental	T11P01A01	C3H/HeN	D7	Skin
ruvA1D7M1S16	Parental	T11P01A01	C3H/HeN	D7	Skin
ruvA1D7M1S17	Parental	T11P01A01	C3H/HeN	D7	Skin
ruvA1D7M1S18	Parental	T11P01A01	C3H/HeN	D7	Skin

ruvA1D7M1S19	Parental	T11P01A01	C3H/HeN	D7	Skin
ruvA1D7M1S20	Parental	T11P01A01	C3H/HeN	D7	Skin
ruvA1D7M1S21	Parental	T11P01A01	C3H/HeN	D7	Skin
ruvA1D7M1S23	Parental	T11P01A01	C3H/HeN	D7	Skin
ruvA1D7M1S24	Parental	T11P01A01	C3H/HeN	D7	Skin
ruvA1D7M1S25	Parental	T11P01A01	C3H/HeN	D7	Skin
ruvA1D7M2S01	Parental	T11P01A01	C3H/HeN	D7	Skin
ruvA1D7M2S02	Parental	T11P01A01	C3H/HeN	D7	Skin
ruvA1D7M2S03	Parental	T11P01A01	C3H/HeN	D7	Skin
ruvA1D7M2S04	Parental	T11P01A01	C3H/HeN	D7	Skin
ruvA1D7M2S05	Parental	T11P01A01	C3H/HeN	D7	Skin
ruvA1D7M2S07	Parental	T11P01A01	C3H/HeN	D7	Skin
ruvA1D7M2S08	Parental	T11P01A01	C3H/HeN	D7	Skin
ruvA1D7M2S09	Parental	T11P01A01	C3H/HeN	D7	Skin
ruvA1D7M2S10	Parental	T11P01A01	C3H/HeN	D7	Skin
ruvA1D7M2S11	Parental	T11P01A01	C3H/HeN	D7	Skin
ruvA1D7M2S12	Parental	T11P01A01	C3H/HeN	D7	Skin
ruvA1D7M2S14	Parental	T11P01A01	C3H/HeN	D7	Skin
ruvA1D7M2S15	Parental	T11P01A01	C3H/HeN	D7	Skin
ruvA1D7M2S16	Parental	T11P01A01	C3H/HeN	D7	Skin
ruvA1D7M2S18	Parental	T11P01A01	C3H/HeN	D7	Skin
ruvA1D7M2S20	Parental	T11P01A01	C3H/HeN	D7	Skin
ruvA1D7M2S21	Parental	T11P01A01	C3H/HeN	D7	Skin
ruvA1D7M2S23	Parental	T11P01A01	C3H/HeN	D7	Skin
ruvA1D7M2S24	Parental	T11P01A01	C3H/HeN	D7	Skin
ruvA1D7M3S01	Parental	T11P01A01	C3H/HeN	D7	Skin
ruvA1D7M3S02	Parental	T11P01A01	C3H/HeN	D7	Skin
ruvA1D7M3S03	Parental	T11P01A01	C3H/HeN	D7	Skin
ruvA1D7M3S04	Parental	T11P01A01	C3H/HeN	D7	Skin
ruvA1D7M3S05	Parental	T11P01A01	C3H/HeN	D7	Skin
ruvA1D7M3S07	Parental	T11P01A01	C3H/HeN	D7	Skin
ruvA1D7M3S08	Parental	T11P01A01	C3H/HeN	D7	Skin
ruvA1D7M3S09	Parental	T11P01A01	C3H/HeN	D7	Skin
ruvA1D7M3S10	Parental	T11P01A01	C3H/HeN	D7	Skin
ruvA1D7M3S11	Parental	T11P01A01	C3H/HeN	D7	Skin
ruvA1D7M3S12	Parental	T11P01A01	C3H/HeN	D7	Skin
ruvA1D7M3S13	Parental	T11P01A01	C3H/HeN	D7	Skin
ruvA1D7M3S14	Parental	T11P01A01	C3H/HeN	D7	Skin
ruvA1D7M3S15	Parental	T11P01A01	C3H/HeN	D7	Skin
ruvA1D7M3S16	Parental	T11P01A01	C3H/HeN	D7	Skin
ruvA1D7M3S17	Parental	T11P01A01	C3H/HeN	D7	Skin
ruvA1D7M3S18	Parental	T11P01A01	C3H/HeN	D7	Skin
ruvA1D7M3S20	Parental	T11P01A01	C3H/HeN	D7	Skin
ruvA1D7M3S21	Parental	T11P01A01	C3H/HeN	D7	Skin
ruvA1D7M3S23	Parental	T11P01A01	C3H/HeN	D7	Skin
ruvA1D7M3S24	Parental	T11P01A01	C3H/HeN	D7	Skin
ruvA1D7M4S02	Parental	T11P01A01	C3H/HeN	D7	Skin

ruvA1D7M4S04	Parental	T11P01A01	C3H/HeN	D7	Skin
ruvA1D7M4S05	Parental	T11P01A01	C3H/HeN	D7	Skin
ruvA1D7M4S06	Parental	T11P01A01	C3H/HeN	D7	Skin
ruvA1D7M4S07	Parental	T11P01A01	C3H/HeN	D7	Skin
ruvA1D7M4S08	Parental	T11P01A01	C3H/HeN	D7	Skin
ruvA1D7M4S09	Parental	T11P01A01	C3H/HeN	D7	Skin
ruvA1D7M4S10	Parental	T11P01A01	C3H/HeN	D7	Skin
ruvA1D7M4S11	Parental	T11P01A01	C3H/HeN	D7	Skin
ruvA1D7M4S12	Parental	T11P01A01	C3H/HeN	D7	Skin
ruvA1D7M4S13	Parental	T11P01A01	C3H/HeN	D7	Skin
ruvA1D7M4S14	Parental	T11P01A01	C3H/HeN	D7	Skin
ruvA1D7M4S15	Parental	T11P01A01	C3H/HeN	D7	Skin
ruvA1D7M4S16	Parental	T11P01A01	C3H/HeN	D7	Skin
ruvA1D7M4S17	Parental	T11P01A01	C3H/HeN	D7	Skin
ruvA1D7M4S18	Parental	T11P01A01	C3H/HeN	D7	Skin
ruvA1D7M4S19	Parental	T11P01A01	C3H/HeN	D7	Skin
ruvA1D7M4S20	Parental	T11P01A01	C3H/HeN	D7	Skin
ruvA1D7M5S01	Parental	T11P01A01	C3H/HeN	D7	Skin
ruvA1D7M5S03	Parental	T11P01A01	C3H/HeN	D7	Skin
ruvA1D7M5S04	Parental	T11P01A01	C3H/HeN	D7	Skin
ruvA1D7M5S05	Parental	T11P01A01	C3H/HeN	D7	Skin
ruvA1D7M5S06	Parental	T11P01A01	C3H/HeN	D7	Skin
ruvA1D7M5S07	Parental	T11P01A01	C3H/HeN	D7	Skin
ruvA1D7M5S08	Parental	T11P01A01	C3H/HeN	D7	Skin
ruvA1D7M5S09	Parental	T11P01A01	C3H/HeN	D7	Skin
ruvA1D7M5S10	Parental	T11P01A01	C3H/HeN	D7	Skin
ruvA1D7M5S11	Parental	T11P01A01	C3H/HeN	D7	Skin
ruvA1D7M5S12	Parental	T11P01A01	C3H/HeN	D7	Skin
ruvA1D7M5S13	Parental	T11P01A01	C3H/HeN	D7	Skin
ruvA1D7M5S14	Parental	T11P01A01	C3H/HeN	D7	Skin
ruvA1D7M5S15	Parental	T11P01A01	C3H/HeN	D7	Skin
ruvA1D7M5S16	Parental	T11P01A01	C3H/HeN	D7	Skin
ruvA1D7M5S17	Parental	T11P01A01	C3H/HeN	D7	Skin
ruvA1D7M5S18	Parental	T11P01A01	C3H/HeN	D7	Skin
ruvA1D7M5S19	Parental	T11P01A01	C3H/HeN	D7	Skin
ruvA1D7M5S20	Parental	T11P01A01	C3H/HeN	D7	Skin
ruvA1D7M5S21	Parental	T11P01A01	C3H/HeN	D7	Skin
ruvA1D7M5S22	Parental	T11P01A01	C3H/HeN	D7	Skin
ruvA1D7M5S23	Parental	T11P01A01	C3H/HeN	D7	Skin
ruvA1D7M5S24	Parental	T11P01A01	C3H/HeN	D7	Skin
ruvA1D14M1J02	Parental	T11P01A01	C3H/HeN	D14	Joint
ruvA1D14M1J03	Parental	T11P01A01	C3H/HeN	D14	Joint
ruvA1D14M1J04	Parental	T11P01A01	C3H/HeN	D14	Joint
ruvA1D14M1J05	Parental	T11P01A01	C3H/HeN	D14	Joint
ruvA1D14M1J06	Parental	T11P01A01	C3H/HeN	D14	Joint
ruvA1D14M1J07	Parental	T11P01A01	C3H/HeN	D14	Joint
ruvA1D14M1J09	Parental	T11P01A01	C3H/HeN	D14	Joint

ruvA1D14M1J10	Parental	T11P01A01	C3H/HeN	D14	Joint
ruvA1D14M2J01	Parental	T11P01A01	C3H/HeN	D14	Joint
ruvA1D14M2J02	Parental	T11P01A01	C3H/HeN	D14	Joint
ruvA1D14M2J05	Parental	T11P01A01	C3H/HeN	D14	Joint
ruvA1D14M2J09	Parental	T11P01A01	C3H/HeN	D14	Joint
ruvA1D14M2J10	Parental	T11P01A01	C3H/HeN	D14	Joint
ruvA1D14M3J05	Parental	T11P01A01	C3H/HeN	D14	Joint
ruvA1D14M3J08	Parental	T11P01A01	C3H/HeN	D14	Joint
ruvA1D14M3J10	Parental	T11P01A01	C3H/HeN	D14	Joint
ruvA1D28M1S04	GQ369323	T11P01A01	C3H/HeN	D28	Skin
ruvA1D28M1S07	GQ369324	T11P01A01	C3H/HeN	D28	Skin
ruvA1D28M1S12	GQ369325	T11P01A01	C3H/HeN	D28	Skin
ruvA1D28M1S14	GQ369326	T11P01A01	C3H/HeN	D28	Skin
ruvA1D28M1S15	GQ369327	T11P01A01	C3H/HeN	D28	Skin
ruvA1D28M1S16	GQ369328	T11P01A01	C3H/HeN	D28	Skin
ruvA1D28M1S17	GQ369329	T11P01A01	C3H/HeN	D28	Skin
ruvA1D28M1S20	GQ369330	T11P01A01	C3H/HeN	D28	Skin
ruvA1D28M1S21	GQ369331	T11P01A01	C3H/HeN	D28	Skin
ruvA1D28M1S22	GQ369332	T11P01A01	C3H/HeN	D28	Skin
ruvA1D28M1S24	GQ369333	T11P01A01	C3H/HeN	D28	Skin
ruvA1D28M1S25	GQ369334	T11P01A01	C3H/HeN	D28	Skin
ruvA1D28M2J01	GQ369386	T11P01A01	C3H/HeN	D28	Joint
ruvA1D28M2J03	GQ369387	T11P01A01	C3H/HeN	D28	Joint
ruvA1D28M2J06	GQ369388	T11P01A01	C3H/HeN	D28	Joint
ruvA1D28M2J08	GQ369389	T11P01A01	C3H/HeN	D28	Joint
ruvA1D28M2J09	GQ369390	T11P01A01	C3H/HeN	D28	Joint
ruvA1D28M2J10	GQ369391	T11P01A01	C3H/HeN	D28	Joint
ruvA1D28M2J11	GQ369392	T11P01A01	C3H/HeN	D28	Joint
ruvA1D28M2J13	GQ369393	T11P01A01	C3H/HeN	D28	Joint
ruvA1D28M2J14	GQ369394	T11P01A01	C3H/HeN	D28	Joint
ruvA1D28M2J16	GQ369395	T11P01A01	C3H/HeN	D28	Joint
ruvA1D28M2J17	GQ369396	T11P01A01	C3H/HeN	D28	Joint
ruvA1D28M2J18	GQ369397	T11P01A01	C3H/HeN	D28	Joint
ruvA1D28M2J19	GQ369398	T11P01A01	C3H/HeN	D28	Joint
ruvA1D28M2J20	GQ369399	T11P01A01	C3H/HeN	D28	Joint
ruvA1D28M2J22	GQ369400	T11P01A01	C3H/HeN	D28	Joint
ruvA1D28M2J23	GQ369401	T11P01A01	C3H/HeN	D28	Joint
ruvA1D28M2J24	GQ369402	T11P01A01	C3H/HeN	D28	Joint
ruvA1D28M3B01	GQ369356	T11P01A01	C3H/HeN	D28	Bladder
ruvA1D28M3B03	GQ369357	T11P01A01	C3H/HeN	D28	Bladder
ruvA1D28M3B04	GQ369358	T11P01A01	C3H/HeN	D28	Bladder
ruvA1D28M3B07	GQ369359	T11P01A01	C3H/HeN	D28	Bladder
ruvA1D28M3B09	GQ369360	T11P01A01	C3H/HeN	D28	Bladder
ruvA1D28M3B10	GQ369361	T11P01A01	C3H/HeN	D28	Bladder
ruvA1D28M3B11	GQ369362	T11P01A01	C3H/HeN	D28	Bladder
ruvA1D28M3B14	GQ369363	T11P01A01	C3H/HeN	D28	Bladder
ruvA1D28M3B15	GQ369364	T11P01A01	C3H/HeN	D28	Bladder

ruvA1D28M3B18	GQ369365	T11P01A01	C3H/HeN	D28	Bladder
ruvA1D28M3B19	GQ369366	T11P01A01	C3H/HeN	D28	Bladder
ruvA1D28M3B20	GQ369367	T11P01A01	C3H/HeN	D28	Bladder
ruvA1D28M3B21	GQ369368	T11P01A01	C3H/HeN	D28	Bladder
ruvA1D28M3B22	GQ369369	T11P01A01	C3H/HeN	D28	Bladder
ruvA1D28M3B24	GQ369370	T11P01A01	C3H/HeN	D28	Bladder
ruvA1D28M3B31	GQ369371	T11P01A01	C3H/HeN	D28	Bladder
ruvA1D28M3B32	GQ369372	T11P01A01	C3H/HeN	D28	Bladder
ruvA1D28M3B33	GQ369373	T11P01A01	C3H/HeN	D28	Bladder
ruvA1D28M3B37	GQ369374	T11P01A01	C3H/HeN	D28	Bladder
ruvA1D28M3B38	GQ369375	T11P01A01	C3H/HeN	D28	Bladder
ruvA1D28M3B39	GQ369376	T11P01A01	C3H/HeN	D28	Bladder
ruvA1D28M3B41	GQ369377	T11P01A01	C3H/HeN	D28	Bladder
ruvA1D28M3B42	GQ369378	T11P01A01	C3H/HeN	D28	Bladder
ruvA1D28M3B44	GQ369379	T11P01A01	C3H/HeN	D28	Bladder
ruvA1D28M3B45	GQ369380	T11P01A01	C3H/HeN	D28	Bladder
ruvA1D28M3B46	GQ369381	T11P01A01	C3H/HeN	D28	Bladder
ruvA1D28M3B47	GQ369382	T11P01A01	C3H/HeN	D28	Bladder
ruvA1D28M3B48	GQ369383	T11P01A01	C3H/HeN	D28	Bladder
ruvA1D28M3B49	GQ369384	T11P01A01	C3H/HeN	D28	Bladder
ruvA1D28M3B51	GQ369385	T11P01A01	C3H/HeN	D28	Bladder
ruvA1D28M3J02	GQ369348	T11P01A01	C3H/HeN	D28	Joint
ruvA1D28M3J08	GQ369349	T11P01A01	C3H/HeN	D28	Joint
ruvA1D28M3J14	GQ369350	T11P01A01	C3H/HeN	D28	Joint
ruvA1D28M3J16	GQ369351	T11P01A01	C3H/HeN	D28	Joint
ruvA1D28M3J19	GQ369352	T11P01A01	C3H/HeN	D28	Joint
ruvA1D28M3J20	GQ369353	T11P01A01	C3H/HeN	D28	Joint
ruvA1D28M3J21	GQ369354	T11P01A01	C3H/HeN	D28	Joint
ruvA1D28M3J23	GQ369355	T11P01A01	C3H/HeN	D28	Joint
ruvA1D28M3S02	GQ369335	T11P01A01	C3H/HeN	D28	Skin
ruvA1D28M3S04	GQ369336	T11P01A01	C3H/HeN	D28	Skin
ruvA1D28M3S05	GQ369337	T11P01A01	C3H/HeN	D28	Skin
ruvA1D28M3S06	GQ369338	T11P01A01	C3H/HeN	D28	Skin
ruvA1D28M3S08	GQ369339	T11P01A01	C3H/HeN	D28	Skin
ruvA1D28M3S09	GQ369340	T11P01A01	C3H/HeN	D28	Skin
ruvA1D28M3S10	GQ369341	T11P01A01	C3H/HeN	D28	Skin
ruvA1D28M3S12	GQ369342	T11P01A01	C3H/HeN	D28	Skin
ruvA1D28M3S15	GQ369343	T11P01A01	C3H/HeN	D28	Skin
ruvA1D28M3S19	GQ369344	T11P01A01	C3H/HeN	D28	Skin
ruvA1D28M3S21	GQ369345	T11P01A01	C3H/HeN	D28	Skin
ruvA1D28M3S22	GQ369346	T11P01A01	C3H/HeN	D28	Skin
ruvA1D28M3S24	GQ369347	T11P01A01	C3H/HeN	D28	Skin
ruvA1D28M4H2	GQ369321	T11P01A01	C3H/HeN	D28	Heart
ruvA1D28M4H24	GQ369312	T11P01A01	C3H/HeN	D28	Heart
ruvA1D28M4H27	GQ369313	T11P01A01	C3H/HeN	D28	Heart
ruvA1D28M4H4	GQ369314	T11P01A01	C3H/HeN	D28	Heart
ruvA1D28M4H5	GQ369315	T11P01A01	C3H/HeN	D28	Heart

ruvA1D28M4H5	GQ369322	T11P01A01	C3H/HeN	D28	Heart
ruvA1D28M4J10	GQ369316	T11P01A01	C3H/HeN	D28	Joint
ruvA1D28M4J3	Parental	T11P01A01	C3H/HeN	D28	Joint
ruvA1D28M4J5	GQ369318	T11P01A01	C3H/HeN	D28	Joint
ruvA1D28M4J8	Parental	T11P01A01	C3H/HeN	D28	Joint
ruvA1D28M4J9	Parental	T11P01A01	C3H/HeN	D28	Joint
ruvA1D28M5H1	GQ369299	T11P01A01	C3H/HeN	D28	Heart
ruvA1D28M5H2	GQ369300	T11P01A01	C3H/HeN	D28	Heart
ruvA1D28M5H5	GQ369301	T11P01A01	C3H/HeN	D28	Heart
ruvA1D28M5H6	GQ369302	T11P01A01	C3H/HeN	D28	Heart
ruvA1D28M5H7	GQ369303	T11P01A01	C3H/HeN	D28	Heart
ruvA1D28M5H8	GQ369304	T11P01A01	C3H/HeN	D28	Heart
ruvA1D28M5H9	GQ369305	T11P01A01	C3H/HeN	D28	Heart
ruvA1D28M5J1	GQ369306	T11P01A01	C3H/HeN	D28	Joint
ruvA1D28M5J3	GQ369307	T11P01A01	C3H/HeN	D28	Joint
ruvA1D28M5J4	GQ369308	T11P01A01	C3H/HeN	D28	Joint
ruvA1D28M5J9	GQ369309	T11P01A01	C3H/HeN	D28	Joint
ruvA1D28M5S1	GQ369310	T11P01A01	C3H/HeN	D28	Skin
ruvA1D28M5S6	GQ369311	T11P01A01	C3H/HeN	D28	Skin
ruvA1D28M6B2	GQ369289	T11P01A01	C3H/HeN	D28	Bladder
ruvA1D28M6B3	GQ369290	T11P01A01	C3H/HeN	D28	Bladder
ruvA1D28M6B4	GQ369291	T11P01A01	C3H/HeN	D28	Bladder
ruvA1D28M6B6	GQ369292	T11P01A01	C3H/HeN	D28	Bladder
ruvA1D28M6B7	GQ369293	T11P01A01	C3H/HeN	D28	Bladder
ruvA1D28M6B8	GQ369294	T11P01A01	C3H/HeN	D28	Bladder
ruvA1D28M6S1	GQ369295	T11P01A01	C3H/HeN	D28	Skin
ruvA1D28M6S4	GQ369296	T11P01A01	C3H/HeN	D28	Skin
ruvA1D28M6S6	GQ369297	T11P01A01	C3H/HeN	D28	Skin
ruvA1D28M6S8	GQ369298	T11P01A01	C3H/HeN	D28	Skin
ruvA1SD14M1B1	Parental	T11P01A01	C3H/scid	D14	Bladder
ruvA1SD14M1B2	Parental	T11P01A01	C3H/scid	D14	Bladder
ruvA1SD14M1B3	Parental	T11P01A01	C3H/scid	D14	Bladder
ruvA1SD14M1B4	Parental	T11P01A01	C3H/scid	D14	Bladder
ruvA1SD14M1B5	Parental	T11P01A01	C3H/scid	D14	Skin
ruvA1SD14M1H1	Parental	T11P01A01	C3H/scid	D14	Heart
ruvA1SD14M1H2	Parental	T11P01A01	C3H/scid	D14	Heart
ruvA1SD14M1H5	Parental	T11P01A01	C3H/scid	D14	Heart
ruvA1SD14M1H6	Parental	T11P01A01	C3H/scid	D14	Heart
ruvA1SD14M1J1	Parental	T11P01A01	C3H/scid	D14	Joint
ruvA1SD14M1J2	Parental	T11P01A01	C3H/scid	D14	Joint
ruvA1SD14M1J3	Parental	T11P01A01	C3H/scid	D14	Joint
ruvA1SD14M1J5	Parental	T11P01A01	C3H/scid	D14	Joint
ruvA1SD14M1S2	Parental	T11P01A01	C3H/scid	D14	Skin
ruvA1SD14M1S5	Parental	T11P01A01	C3H/scid	D14	Skin
ruvA1SD14M1S6	Parental	T11P01A01	C3H/scid	D14	Skin
ruvA1SD14M2B1	Parental	T11P01A01	C3H/scid	D14	Bladder
ruvA1SD14M2B2	Parental	T11P01A01	C3H/scid	D14	Bladder

ruvA1SD14M2B4	Parental	T11P01A01	C3H/scid	D14	Bladder
ruvA1SD14M2B6	Parental	T11P01A01	C3H/scid	D14	Bladder
ruvA1SD14M2H2	Parental	T11P01A01	C3H/scid	D14	Heart
ruvA1SD14M2H4	Parental	T11P01A01	C3H/scid	D14	Heart
ruvA1SD14M2H5	Parental	T11P01A01	C3H/scid	D14	Heart
ruvA1SD14M2H6	Parental	T11P01A01	C3H/scid	D14	Heart
ruvA1SD14M2J1	Parental	T11P01A01	C3H/scid	D14	Joint
ruvA1SD14M2J2	Parental	T11P01A01	C3H/scid	D14	Joint
ruvA1SD14M2J3	Parental	T11P01A01	C3H/scid	D14	Joint
ruvA1SD14M2J4	Parental	T11P01A01	C3H/scid	D14	Joint
ruvA1SD14M2J5	Parental	T11P01A01	C3H/scid	D14	Joint
ruvA1SD14M2J6	Parental	T11P01A01	C3H/scid	D14	Joint
ruvA1SD14M2S2	Parental	T11P01A01	C3H/scid	D14	Skin
ruvA1SD14M2S4	Parental	T11P01A01	C3H/scid	D14	Skin
ruvA1SD14M2S5	Parental	T11P01A01	C3H/scid	D14	Skin
ruvA1SD14M2S6	Parental	T11P01A01	C3H/scid	D14	Skin
ruvA1SD14M3B1	Parental	T11P01A01	C3H/scid	D14	Bladder
ruvA1SD14M3B2	Parental	T11P01A01	C3H/scid	D14	Bladder
ruvA1SD14M3B4	Parental	T11P01A01	C3H/scid	D14	Bladder
ruvA1SD14M3B5	Parental	T11P01A01	C3H/scid	D14	Bladder
ruvA1SD14M3B6	Parental	T11P01A01	C3H/scid	D14	Bladder
ruvA1SD14M3H1	Parental	T11P01A01	C3H/scid	D14	Heart
ruvA1SD14M3H2	Parental	T11P01A01	C3H/scid	D14	Heart
ruvA1SD14M3H3	Parental	T11P01A01	C3H/scid	D14	Heart
ruvA1SD14M3H5	Parental	T11P01A01	C3H/scid	D14	Heart
ruvA1SD14M3H6	Parental	T11P01A01	C3H/scid	D14	Heart
ruvA1SD14M3J1	Parental	T11P01A01	C3H/scid	D14	Joint
ruvA1SD14M3J3	Parental	T11P01A01	C3H/scid	D14	Joint
ruvA1SD14M3J4	Parental	T11P01A01	C3H/scid	D14	Joint
ruvA1SD14M3J5	Parental	T11P01A01	C3H/scid	D14	Joint
ruvA1SD14M3J6	Parental	T11P01A01	C3H/scid	D14	Joint
ruvA1SD14M3S1	Parental	T11P01A01	C3H/scid	D14	Skin
ruvA1SD14M3S4	Parental	T11P01A01	C3H/scid	D14	Skin
ruvA1SD14M3S5	Parental	T11P01A01	C3H/scid	D14	Skin
ruvA1SD14M3S6	Parental	T11P01A01	C3H/scid	D14	Skin
ruvA1SD14M4B2	Parental	T11P01A01	C3H/scid	D14	Bladder
ruvA1SD14M4B3	Parental	T11P01A01	C3H/scid	D14	Bladder
ruvA1SD14M4B4	Parental	T11P01A01	C3H/scid	D14	Bladder
ruvA1SD14M4H3	Parental	T11P01A01	C3H/scid	D14	Heart
ruvA1SD14M4H6	Parental	T11P01A01	C3H/scid	D14	Heart
ruvA1SD14M4J1	Parental	T11P01A01	C3H/scid	D14	Joint
ruvA1SD14M4J3	Parental	T11P01A01	C3H/scid	D14	Joint
ruvA1SD14M4J4	Parental	T11P01A01	C3H/scid	D14	Joint
ruvA1SD14M4J5	Parental	T11P01A01	C3H/scid	D14	Joint
ruvA1SD14M4J6	Parental	T11P01A01	C3H/scid	D14	Joint
ruvA1SD14M4S1	Parental	T11P01A01	C3H/scid	D14	Skin
ruvA1SD14M4S2	Parental	T11P01A01	C3H/scid	D14	Skin

ruvA1SD14M4S3	Parental	T11P01A01	C3H/scid	D14	Skin
ruvA1SD14M4S4	Parental	T11P01A01	C3H/scid	D14	Skin
ruvA1SD14M4S5	Parental	T11P01A01	C3H/scid	D14	Skin
ruvA1SD14M4S6	Parental	T11P01A01	C3H/scid	D14	Skin
ruvA1SD14M5B1	Parental	T11P01A01	C3H/scid	D14	Bladder
ruvA1SD14M5B3	Parental	T11P01A01	C3H/scid	D14	Bladder
ruvA1SD14M5B5	Parental	T11P01A01	C3H/scid	D14	Bladder
ruvA1SD14M5H2	Parental	T11P01A01	C3H/scid	D14	Heart
ruvA1SD14M5H3	Parental	T11P01A01	C3H/scid	D14	Heart
ruvA1SD14M5H5	Parental	T11P01A01	C3H/scid	D14	Heart
ruvA1SD14M5J1	Parental	T11P01A01	C3H/scid	D14	Joint
ruvA1SD14M5J3	Parental	T11P01A01	C3H/scid	D14	Joint
ruvA1SD14M5J4	Parental	T11P01A01	C3H/scid	D14	Joint
ruvA1SD14M5J5	Parental	T11P01A01	C3H/scid	D14	Joint
ruvA1SD14M5S1	Parental	T11P01A01	C3H/scid	D14	Skin
ruvA1SD14M5S2	Parental	T11P01A01	C3H/scid	D14	Skin
ruvA1SD14M5S3	Parental	T11P01A01	C3H/scid	D14	Skin
ruvA1SD14M5S4	Parental	T11P01A01	C3H/scid	D14	Skin
ruvA1SD14M5S5	Parental	T11P01A01	C3H/scid	D14	Skin
ruvA1SD14M5S6	Parental	T11P01A01	C3H/scid	D14	Skin
ruvA1SD28M1B1	Parental	T11P01A01	C3H/scid	D28	Bladder
ruvA1SD28M1B3	Parental	T11P01A01	C3H/scid	D28	Bladder
ruvA1SD28M1B4	Parental	T11P01A01	C3H/scid	D28	Bladder
ruvA1SD28M1B6	Parental	T11P01A01	C3H/scid	D28	Bladder
ruvA1SD28M1H1	Parental	T11P01A01	C3H/scid	D28	Heart
ruvA1SD28M1H2	Parental	T11P01A01	C3H/scid	D28	Heart
ruvA1SD28M1H2	Parental	T11P01A01	C3H/scid	D28	Heart
ruvA1SD28M1H4	Parental	T11P01A01	C3H/scid	D28	Heart
ruvA1SD28M1H4	Parental	T11P01A01	C3H/scid	D28	Heart
ruvA1SD28M1H5	Parental	T11P01A01	C3H/scid	D28	Heart
ruvA1SD28M1H6	Parental	T11P01A01	C3H/scid	D28	Heart
ruvA1SD28M1H6	Parental	T11P01A01	C3H/scid	D28	Heart
ruvA1SD28M1J6	Parental	T11P01A01	C3H/scid	D28	Joint
ruvA1SD28M1S1	Parental	T11P01A01	C3H/scid	D28	Skin
ruvA1SD28M1S2	Parental	T11P01A01	C3H/scid	D28	Skin
ruvA1SD28M1S3	Parental	T11P01A01	C3H/scid	D28	Skin
ruvA1SD28M1S3	Parental	T11P01A01	C3H/scid	D28	Skin
ruvA1SD28M1S4	Parental	T11P01A01	C3H/scid	D28	Skin
ruvA1SD28M1S5	Parental	T11P01A01	C3H/scid	D28	Skin
ruvA1SD28M1S5	Parental	T11P01A01	C3H/scid	D28	Skin
ruvA1SD28M1S6	Parental	T11P01A01	C3H/scid	D28	Skin
ruvA1SD28M1S6	Parental	T11P01A01	C3H/scid	D28	Skin
ruvA1SD28M2B3	Parental	T11P01A01	C3H/scid	D28	Bladder
ruvA1SD28M2B4	Parental	T11P01A01	C3H/scid	D28	Bladder
ruvA1SD28M2J1	Parental	T11P01A01	C3H/scid	D28	Joint
ruvA1SD28M2J2	Parental	T11P01A01	C3H/scid	D28	Joint
ruvA1SD28M2J3	Parental	T11P01A01	C3H/scid	D28	Joint

ruvA1SD28M2J4	Parental	T11P01A01	C3H/scid	D28	Joint
ruvA1SD28M2J6	Parental	T11P01A01	C3H/scid	D28	Joint
ruvA1SD28M2S1	Parental	T11P01A01	C3H/scid	D28	Skin
ruvA1SD28M2S2	Parental	T11P01A01	C3H/scid	D28	Skin
ruvA1SD28M2S3	Parental	T11P01A01	C3H/scid	D28	Skin
ruvA1SD28M3B3	Parental	T11P01A01	C3H/scid	D28	Bladder
ruvA1SD28M3B4	Parental	T11P01A01	C3H/scid	D28	Bladder
ruvA1SD28M3B5	Parental	T11P01A01	C3H/scid	D28	Bladder
ruvA1SD28M3H4	Parental	T11P01A01	C3H/scid	D28	Heart
ruvA1SD28M3J1	Parental	T11P01A01	C3H/scid	D28	Joint
ruvA1SD28M3J2	Parental	T11P01A01	C3H/scid	D28	Joint
ruvA1SD28M3J3	Parental	T11P01A01	C3H/scid	D28	Joint
ruvA1SD28M3J4	Parental	T11P01A01	C3H/scid	D28	Joint
ruvA1SD28M3J5	Parental	T11P01A01	C3H/scid	D28	Joint
ruvA1SD28M3J6	Parental	T11P01A01	C3H/scid	D28	Joint
ruvA1SD28M3J7	Parental	T11P01A01	C3H/scid	D28	Joint
ruvA1SD28M3J8	Parental	T11P01A01	C3H/scid	D28	Joint
ruvA1SD28M3S2	Parental	T11P01A01	C3H/scid	D28	Skin
ruvA1SD28M4B4	Parental	T11P01A01	C3H/scid	D28	Bladder
ruvA1SD28M4J1	Parental	T11P01A01	C3H/scid	D28	Joint
ruvA1SD28M4J6	Parental	T11P01A01	C3H/scid	D28	Joint
ruvA1SD28M4S3	Parental	T11P01A01	C3H/scid	D28	Skin
ruvA1SD28M4S4	Parental	T11P01A01	C3H/scid	D28	Skin
ruvA1SD28M5B1	Parental	T11P01A01	C3H/scid	D28	Bladder
ruvA1SD28M5B3	Parental	T11P01A01	C3H/scid	D28	Bladder
ruvA1SD28M5B4	Parental	T11P01A01	C3H/scid	D28	Bladder
ruvA1SD28M5B6	Parental	T11P01A01	C3H/scid	D28	Bladder
ruvA1SD28M5H2	Parental	T11P01A01	C3H/scid	D28	Heart
ruvA1SD28M5H3	Parental	T11P01A01	C3H/scid	D28	Heart
ruvA1SD28M5H5	Parental	T11P01A01	C3H/scid	D28	Heart
ruvA1SD28M5H6	Parental	T11P01A01	C3H/scid	D28	Heart
ruvA1SD28M5J2	Parental	T11P01A01	C3H/scid	D28	Joint
ruvA1SD28M5J3	Parental	T11P01A01	C3H/scid	D28	Joint
ruvA1SD28M5J4	Parental	T11P01A01	C3H/scid	D28	Joint
ruvA1SD28M5J5	Parental	T11P01A01	C3H/scid	D28	Joint
ruvA1SD28M5J6	Parental	T11P01A01	C3H/scid	D28	Joint
ruvA1SD28M5J7	Parental	T11P01A01	C3H/scid	D28	Joint
ruvA1SD28M5S2	Parental	T11P01A01	C3H/scid	D28	Skin
ruvA1SD28M5S4	Parental	T11P01A01	C3H/scid	D28	Skin
ruvA1SD28M5S5	Parental	T11P01A01	C3H/scid	D28	Skin
ruvA1SD28M5S6	Parental	T11P01A01	C3H/scid	D28	Skin
ruvB1D7M1S3	Parental	T03TC051	C3H/HeN	D7	Skin
ruvB1D7M1S5	Parental	T03TC051	C3H/HeN	D7	Skin
ruvB1D7M1S7	Parental	T03TC051	C3H/HeN	D7	Skin
ruvB1D7M1S8	Parental	T03TC051	C3H/HeN	D7	Skin
ruvB1D7M2S1	Parental	T03TC051	C3H/HeN	D7	Skin
ruvB1D7M2S2	Parental	T03TC051	C3H/HeN	D7	Skin

ruvB1D7M2S4	Parental	T03TC051	C3H/HeN	D7	Skin
ruvB1D7M2S5	Parental	T03TC051	C3H/HeN	D7	Skin
ruvB1D7M2S6	Parental	T03TC051	C3H/HeN	D7	Skin
ruvB1D7M2S7	Parental	T03TC051	C3H/HeN	D7	Skin
ruvB1D7M2S8	Parental	T03TC051	C3H/HeN	D7	Skin
ruvB1D7M3S4	Parental	T03TC051	C3H/HeN	D7	Skin
ruvB1D7M3S5	Parental	T03TC051	C3H/HeN	D7	Skin
ruvB1D7M3S6	Parental	T03TC051	C3H/HeN	D7	Skin
ruvB1D7M3S7	Parental	T03TC051	C3H/HeN	D7	Skin
ruvB1D7M4S2	Parental	T03TC051	C3H/HeN	D7	Skin
ruvB1D7M4S3	Parental	T03TC051	C3H/HeN	D7	Skin
ruvB1D7M4S4	Parental	T03TC051	C3H/HeN	D7	Skin
ruvB1D7M4S5	Parental	T03TC051	C3H/HeN	D7	Skin
ruvB1D7M4S6	Parental	T03TC051	C3H/HeN	D7	Skin
ruvB1D7M4S7	Parental	T03TC051	C3H/HeN	D7	Skin
ruvB1D7M4S8	Parental	T03TC051	C3H/HeN	D7	Skin
ruvB1D28M1B7	GU062653	T03TC051	C3H/HeN	D28	Bladder
ruvB1D28M1H2	GU062654	T03TC051	C3H/HeN	D28	Heart
ruvB1D28M1H3	GU062655	T03TC051	C3H/HeN	D28	Heart
ruvB1D28M1H4	GU062656	T03TC051	C3H/HeN	D28	Heart
ruvB1D28M1H5	GU062657	T03TC051	C3H/HeN	D28	Heart
ruvB1D28M1H6	GU062658	T03TC051	C3H/HeN	D28	Heart
ruvB1D28M1H7	GU062659	T03TC051	C3H/HeN	D28	Heart
ruvB1D28M1J3	GU062660	T03TC051	C3H/HeN	D28	Joint
ruvB1D28M1J4	GU062661	T03TC051	C3H/HeN	D28	Joint
ruvB1D28M1J5	GU062662	T03TC051	C3H/HeN	D28	Joint
ruvB1D28M1S4	GU062663	T03TC051	C3H/HeN	D28	Skin
ruvB1D28M1S5	GU062664	T03TC051	C3H/HeN	D28	Skin
ruvB1D28M1S6	GU062665	T03TC051	C3H/HeN	D28	Skin
ruvB1D28M1S7	GU062666	T03TC051	C3H/HeN	D28	Skin
ruvB1D28M2B1	GU062667	T03TC051	C3H/HeN	D28	Bladder
ruvB1D28M2B2	GU062668	T03TC051	C3H/HeN	D28	Bladder
ruvB1D28M2B4	GU062669	T03TC051	C3H/HeN	D28	Bladder
ruvB1D28M2B5	GU062670	T03TC051	C3H/HeN	D28	Bladder
ruvB1D28M2J1	GU062671	T03TC051	C3H/HeN	D28	Joint
ruvB1D28M2J2	GU062672	T03TC051	C3H/HeN	D28	Joint
ruvB1D28M2J3	GU062673	T03TC051	C3H/HeN	D28	Joint
ruvB1D28M2J5	GU062674	T03TC051	C3H/HeN	D28	Joint
ruvB1D28M2J7	GU062675	T03TC051	C3H/HeN	D28	Joint
ruvB1D28M2S2	GU062676	T03TC051	C3H/HeN	D28	Skin
ruvB1D28M2S3	GU062677	T03TC051	C3H/HeN	D28	Skin
ruvB1D28M2S5	GU062678	T03TC051	C3H/HeN	D28	Skin
ruvB1D28M2S6	GU062679	T03TC051	C3H/HeN	D28	Skin
ruvB1D28M2S8	GU062680	T03TC051	C3H/HeN	D28	Skin
ruvB1D28M3H2	GU062641	T03TC051	C3H/HeN	D28	Heart
ruvB1D28M3H3	GU062642	T03TC051	C3H/HeN	D28	Heart
ruvB1D28M3H5	GU062643	T03TC051	C3H/HeN	D28	Heart

ruvB1D28M3H6	GU062644	T03TC051	C3H/HeN	D28	Heart
ruvB1D28M3H7	GU062645	T03TC051	C3H/HeN	D28	Heart
ruvB1D28M3J2	Parental	T03TC051	C3H/HeN	D28	Joint
ruvB1D28M3J3	Parental	T03TC051	C3H/HeN	D28	Joint
ruvB1D28M3J4	Parental	T03TC051	C3H/HeN	D28	Joint
ruvB1D28M3J5	Parental	T03TC051	C3H/HeN	D28	Joint
ruvB1D28M3J6	Parental	T03TC051	C3H/HeN	D28	Joint
ruvB1D28M3S5	GU062651	T03TC051	C3H/HeN	D28	Skin
ruvB1D28M3S8	GU062652	T03TC051	C3H/HeN	D28	Skin
